# Relational care in community mental health: Evaluating staff experiences in Intensive Community Care Services (ICCS) vs treatment as usual

**DOI:** 10.1371/journal.pone.0356608

**Published:** 2026-09-09

**Authors:** Opeyemi Atanda, Paula Reavey, Ben Wong, Ebony Baker, Jessica Collier, Thilipan Thaventhiran, Veronika Dobler, Ruth Woolhouse, Toby Zundel, Joe Clacey, Jovanka Tolmac, Izabela Pilecka, Leon Wehncke, Tauseef Mehdi, Rhys Bevan-Jones, Dennis Ougrin

**Affiliations:** 1 London South Bank University, London, United Kingdom; 2 East London NHS Foundation Trust, London, United Kingdom; 3 Queen Mary University of London, London, United Kingdom; 4 Cambridge University Hospitals NHS Foundation Trust, Cambridge, United Kingdom; 5 South London and Maudsley NHS Foundation Trust, London, United Kingdom; 6 Oxford University Hospitals NHS Trust, Oxford, United Kingdom; 7 Central and Northwest London NHS Foundation Trust, London, United Kingdom; 8 Kings College London, London, United Kingdom; 9 Northeast London NHS Foundation Trust, London, United Kingdom; 10 Royal Berkshire NHS Foundation Trust, Reading, United Kingdom; 11 Cwm Taf Morgannwg University Health Board, Abercynon, United Kingdom; Amrita Vishwa Vidyapeetham - Amritapuri Campus, INDIA

## Abstract

**Background:**

The quality of healthcare delivery relies heavily on building strong relationships between healthcare providers (HCPs) and clients. This study presents the results of a process evaluation for a Randomised Controlled Trial (RCT) examining the effectiveness of Intensive Community Care Service (ICCS) vs Treatment as Usual (TAU; inpatient or core community CAMHS).

**Methods:**

Thirty-four semi-structured interviews were conducted with staff across various services, including 20 from ICCS and 14 other TAU services. A thematic decomposition analysis was conducted on the data, and specific themes relevant to staff experiences of young people’s engagement with services and overall recovery.

**Results:**

Three main themes were observed in the HCP data (1. Relational Ecologies: barriers and enablers to engagement, 2. flexibility of approach amidst systemic pressures and 3. the web of trust in the relationship-building process). HCPs highlighted the necessity of developing trust and rapport through non-clinical engagement strategies, such as informal visits and personalised interactions. HCPs emphasised that without trust, treatment effectiveness diminishes, necessitating a tailored approach rather than a one-size-fits-all model. The flexibility in duration of treatment and methods of engagement was noted as crucial in accommodating individual client needs and fostering an open, trusting environment necessary for long-term recovery.

**Conclusion:**

The findings highlight the vital importance of relational care models, especially ICCS, in addressing the complex needs of Children and Young People (CYP). Flexible, family-centred approaches improve trust, engagement, and long-term recovery outcomes. Recommendations include tackling systemic barriers and expanding relational care models within CAMHS to meet increasing mental health demands effectively. Further research should investigate scalable strategies for integrating these insights into wider mental health service frameworks.

## Background

The prevalence of mental health challenges among children and young people (CYP) in high-income countries has increased steadily since 1995. Although Child and Adolescent Mental Health Services (CAMHS) have expanded, demand continues to exceed capacity [1]. Inpatient care has traditionally served as the main pathway for severe mental health conditions. However, recent evidence highlights the effectiveness of community-based alternatives in managing clinical risk and need outside institutional settings. Intensive Community Care Services (ICCS) have emerged as an alternative to hospital admission for CYP at high risk [2]. Expert consensus indicates that these approaches improve functional outcomes, reduce admissions, and preserve continuity in home and educational contexts [3]. Intensive Community Care Services (ICCS) are designed to provide an intensive, multidisciplinary alternative to inpatient admission for children and young people experiencing severe mental health difficulties. Unlike conventional outpatient CAMHS provision, ICCS delivers flexible, community-based interventions that extend beyond clinic settings to include home visits, school-based support and wider community engagement. Care is delivered by multidisciplinary teams comprising psychiatrists, psychologists, nurses, occupational therapists, social workers and other allied professionals who work collaboratively to provide coordinated, individualised care. In contrast, Treatment as Usual (TAU) within CAMHS generally consists of clinic-based multidisciplinary care delivered through scheduled outpatient appointments, with the intensity of contact determined by service capacity and clinical need. While both service models aim to deliver evidence-based mental healthcare, ICCS seeks to extend relational care through greater flexibility, continuity and community-based engagement. A summary of the principal characteristics of both service models is provided in Table 1.

**Table 1:** Comparison of key characteristics of Intensive Community Care Services (ICCS) and Treatment as Usual (TAU).

	ICCS	TAU
**Primary setting**	Home, school, community and clinic	Primarily clinic-based.
**Team composition**	Multidisciplinary team (e.g., psychiatry, psychology, nursing, occupational therapy, social work).	Multidisciplinary CAMHS team.
**Intensity of contact**	Flexible and responsive; frequency varies according to need.	Scheduled outpatient appointments based on clinical need and service capacity.
**Family involvement**	Active partnership through collaborative care planning, psychoeducation and ongoing support.	Family involvement according to routine CAMHS practice.
**Approach to engagement**	Assertive outreach and flexible engagement across settings.	Predominantly appointment-based engagement.
**Care planning**	Individualised, multidisciplinary and responsive to changing needs.	Individualised within standard CAMHS care pathways.
**Overall focus**	Intensive community-based support aimed at reducing hospital admission and promoting recovery in natural environments.	Evidence-based outpatient assessment and treatment within routine CAMHS services.

Evidence from adult and youth services supports the value of intensive community models. Assertive Community Treatment (ACT) reduces hospital use and improves stability, symptom management, and life satisfaction [4,5]. Housing First, often combined with ACT, improves housing retention, quality of life, and recovery [6]. Functional ACT (FACT) enhances patient empowerment [7], while Interpersonal Community Psychiatric Treatment (ICPT) demonstrates effectiveness and cost-efficiency for long-term non-psychotic disorders [8]. Reviews show that home treatment, multisystemic therapy, and supported discharge achieve outcomes comparable or superior to inpatient care, while reducing costs and hospital stays and improving satisfaction [9]. These findings support a shift towards rights-based, recovery-oriented, community-centred care aligned with service-user preferences [10–12].

Research consistently shows that many CYP experience inpatient settings as restrictive and distressing [13,14]. Involuntary admissions associate with poorer experiences, higher suicide risk, dissatisfaction, increased readmission, and inequitable treatment of marginalised groups [13]. Post-discharge improvements tend to be modest, and staff evaluations of inpatient environments are often unfavourable [15]. These limitations have accelerated interest in community alternatives.

Emerging evidence indicates that community services can support diverse needs effectively. Forward Thinking Birmingham, developed from Youthspace, integrated assessment and treatment for young people aged 0–25. The model improved access, distributed clinical responsibility, and used digital platforms to enhance engagement. Although crisis and home treatment teams increased service reach, outcome improvements could not be confirmed due to data limitations [16]. This example illustrates both the potential and evaluation challenges of large-scale community initiatives.

Community-based organisations (CBOs) play a critical role in supporting marginalised populations. They provide safe spaces for identity expression, empowerment, and collective action, contributing to improved mental health among low-income, immigrant, and racially minoritised groups [17]. Community approaches for CYP promote personalised care, enhance engagement, and situate support within familiar environments, reducing stigma and encouraging help-seeking [18,19]. Their preventative focus enables early intervention and resilience-building before crises escalate [19].

Young people consistently identify continuity of care as central to therapeutic progress. Community settings often preserve continuity through more stable provider–service-user relationships [20–22]. Integration with local resources strengthens holistic support, and lower readmission rates further demonstrate effectiveness [13]. Cultural competence and family involvement improve relevance and responsiveness [14]. Addressing structural barriers and wider determinants of mental health, including poverty, housing, and social support, remains essential for sustainable prevention [23].

Despite this evidence, recent policy directions have deprioritised structural and community-level factors, even though they contribute substantially to psychological distress [20]. Community-based care re-centres these influences and facilitates contextually informed intervention [14]. Group therapy has also gained prominence, with evidence of reduced self-harm and suicidal ideation and lower crisis service use among adolescents [24]. Although NHS implementation plans prioritise community care for adults with severe mental illness [25], evidence on translating these models to CYP remains limited.

ICCS represents a targeted community-based alternative for CYP at risk of admission. Services typically operate in homes or schools, allowing young people to maintain daily routines while receiving intensive support [2]. Although the evidence base remains limited, existing studies suggest ICCS offers a viable alternative to treatment as usual, including during acute crises [15,26].

Although the wider randomised controlled trial evaluated the effectiveness of Intensive Community Care Services (ICCS) compared with Treatment as Usual (TAU) [27], less is known about how healthcare professionals experience delivering these models of care in routine practice. Understanding these experiences is important because implementation, therapeutic relationships and organisational contexts can influence intervention delivery and sustainability.

Therefore, the present qualitative study aimed to explore healthcare professionals’ experiences of delivering ICCS and TAU within Child and Adolescent Mental Health Services (CAMHS), with particular attention to the relational, organisational and contextual factors that shape engagement, care delivery and recovery.

## Method

This study reports the qualitative component of a mixed-methods, NIHR-funded randomised controlled trial across ten NHS trusts in England and Wales, collecting data on CYP at risk of and eligible for Tier 4 inpatient care (specialist inpatient mental health services), and evaluating ICCS compared with TAU. The qualitative study explored healthcare professionals’ experiences of delivering care within the two service models.. The trial compared ICCS with TAU to assess functioning, self-harm, education, and quality of life. Interviews evaluated perceived effectiveness, emotions, and impact on mental health for staff and service users.

A purposive sampling strategy was used to recruit healthcare professionals involved in delivering either Intensive Community Care Services (ICCS) or Treatment as Usual (TAU) within participating Child and Adolescent Mental Health Services (CAMHS). Purposive sampling was chosen to ensure that participants had direct experience delivering care within the service models under investigation and to represent a range of professional disciplines, including psychiatrists, psychologists, nurses, occupational therapists, and other members of the multidisciplinary team. This approach aimed to capture diverse perspectives across different service contexts.

Sample size in qualitative research is guided by the depth and richness of the data rather than statistical representativeness. Recruitment continued until the research team judged that sufficient information power had been achieved, whereby the data provided adequate breadth and depth to address the study aims [28]. Given the relatively focused research question, the specificity of the participant group and the rich experiential accounts obtained, the final sample was considered sufficient to generate meaningful thematic insights.

Thirty-five semi-structured interviews were conducted with HCP across various services, including intensive community care services (ICCS) (n = 20) and other TAU services (Inpatient (n = 4) & core CAMHS (n = 10)). The professional role, years of experience and service type are summarised in Table 2.

**Table 2:** Characteristics of healthcare professional participants.

Profession	Years of Experience*	Current Service Type
Lead Mental Health Nurse	25	Enhanced Treatment Team (CAMHS)
Support Worker	2	Intensive Community Care Service (ICCS)
Consultant Psychiatrist	3 (consultant)	Crisis Team
Consultant Psychiatrist	8	ICCS & Crisis Team
Mental Health Nurse	~28	Home Treatment Team
Speciality Doctor (Psychiatry)	11	Enhanced Treatment & Emotional Disorders Team
Clinical Psychologist	1.5	Enhanced Treatment Service
Mental Health Nurse / DBT Lead	4	Outreach & DBT Service
Consultant Psychiatrist	12	Tier 4 Inpatient CAMHS
Senior Social Worker	20+	Crisis Team & ICCS
Crisis & Home Treatment Manager	10	Crisis & Home Treatment
Senior Mental Health Practitioner	1	INTERACT Crisis Service
Youth Intensive Psychological Practitioner	2	Home Treatment & Inpatient CAMHS
Consultant Child & Adolescent Psychiatrist	5	Urgent Care Team
Clinical Specialist Practitioner	5	Hospital at Home/Home Treatment
STR Worker / Nursing Associate	11	Home Treatment Team
Mental Health Practitioner	6	Crisis Team & Emotional Behaviour Team
Family Therapist	2 (current service)	Home Treatment Team
Clinical Team Lead	9	ICCS
Mental Health Practitioner	3	Rapid Response Team
Band 7 Mental Health Nurse	0.75	ICCS
Clinical Psychologist	9	Inpatient CAMHS
Support Worker	0.5	Enhanced Treatment Service
Mental Health Practitioner	1.2	Tier 3 CAMHS (formerly ETS)
Lead Consultant Psychiatrist	2	Crisis Team (ICCS model)
Consultant Psychiatrist	9.5+	Enhanced Treatment Service
Crisis Specialist Practitioner	1.5	Urgent Care Outreach Service
Mental Health Nurse	14	Community Intensive Team
Mental Health Practitioner	2	Crisis Treatment Team
Consultant Psychiatrist	7	Liaison, Crisis & Outreach Team
Consultant Psychiatrist / Clinical Research Fellow	3.5	Generic CAMHS
Trainee Youth Intensive Psychological Practitioner	1.5	Home Treatment Team
Senior Nurse & Clinical Team Lead	4	CAMHS Crisis Service
Senior Mental Health Practitioner (Nurse)	24	Home Treatment Team

Recruitment of HCPs for interviews was conducted from 04/12/2022 to 30/01/2024. Clinical leads within participating services identified eligible staff and distributed study information. Interested healthcare professionals contacted the research team directly or gave permission to be contacted. Recruitment continued throughout the qualitative data collection period to maximise variation in professional role and service setting.

Each interview lasted between 45 and 60 minutes. Interviews were audio-recorded with participant consent and transcribed verbatim. Before access to the HCPs was permitted, permission was granted by all local NHS, King’s College London and London South Bank University research ethics committees. Written and verbal informed consent was given by all interviewed participants. Interviews were semi-structured and constructed with a specific set of themes derived from gaps in the literature around the contextual experiences of HCP delivering services in CAMHS, more significantly, the ICCS intervention. However, sufficient flexibility in the interview structure was ensured to prompt participants to elaborate further on their experiences, representing a greater depth of response. The interviews were transcribed verbatim, and the interview guide was developed based on input from a public patient involvement (PPI) group comprising young people who had previously used inpatient and core CAMHS community services. The interview guide is included as a supporting information S1 File.

### Ethical approval

Ethical approval was obtained from the West Midlands and Black Country Research Ethics Committee and the Health Research Authority in the UK, REC reference: 20/WM/0069. The trial was conducted in compliance with the principles of the Declaration of Helsinki (1996), the principles of GCP and in accordance with all applicable regulatory requirements including but not limited to the UK policy framework for health and social care research. The Sponsor trust granted Local Research and Development approval. Informed consent was obtained from all participants.

### Analysis

The validity of the findings was addressed using conventional qualitative procedures, including group analysis by key researchers and peer review, to ensure the analysis was sufficiently grounded in the data [29].

All authors participated collaboratively in the analytical process. Based on the aforementioned questions, the interview material was notated and coded, and the data were restructured into themes following an extensive round of discussion by the research team. This reorganised data was then examined alongside relevant literature to provide a contextual framework for the analysis. A thematic decomposition [30] approach was used to analyse the data thematically [14]. Furthermore, the aim was to identify processes through which mental health service use was enacted, understood, and experienced. This thematic decomposition was achieved by following several stages of analysis commonly found across all qualitative analysis analyses [31] including familiarisation with the data via repeated readings of the transcripts, generating initial codes by paying close attention to meanings embedded in every line of the participants interviews. Initial inductive coding was conducted independently, line by line, by three authors (OA, EB & JC). Codes were generated from meaningful units of text relating to staff experiences of engagement, relationships, service delivery, and recovery. Similar codes were subsequently grouped into candidate themes through iterative discussion among the research team. Coding was managed using NVivo 14 (QSR International) to support systematic organisation and retrieval of the data. The software was used to facilitate data management and coding but did not determine the analytical interpretation. Each of the authors participated in discussions around whether the generated theme titles and definitions adequately captured the essence of the data. There was cross-validation at all analytical stages, including initial data coding, the expansion of coding into themes and the discussion of themes using key data. The interpretative process further involved exploring the implicit meaning of the material by prioritising a deeper understanding of the participants’ experiences rather than a more descriptive reading.

## Findings

Before discussing the main themes of this paper, we provide an overview of the general perspectives of the HCPs’ experience across several services to provide a broader background context to the analysis.

### General overview of services from the perspective of HCPs

HCPs presented a range of their experiences across the services, particularly the ICCS, core CAMHS (TAU), and inpatient wards, reflecting both positive and negative aspects of care. ICCS is designed to provide flexible, community-based mental health support in environments such as schools or homes, enabling young people to maintain their daily routines while receiving care. This approach prioritises trust-building through informal engagement, like casual conversations or games, and relies on a single clinician for continuity, fostering stronger relationships with CYPs and their families. Family involvement is central to ICCS, with psychoeducation and empowerment initiatives helping parents regain autonomy while supporting their child’s recovery. HCPs emphasised the importance of a tailored, culturally sensitive approach that adapts to the individual needs of service users, contrasting with the often-rigid frameworks of TAU. While TAU, which includes inpatient and Core CAHMS services, offers structured interventions, it faces challenges such as long waiting lists, reliance on medication before therapy, and limited opportunities for personalisation, often leaving CYP dissatisfied with their care [14].

Across both ICCS and TAU, healthcare professionals underscored the critical role of trust and engagement in achieving positive outcomes. Barriers such as systemic pressures, resource limitations, and lengthy wait times were identified as significant challenges, particularly in TAU services, which can hinder comprehensive therapeutic interventions. HCPs noted that ICCS’s flexibility in scheduling, location, and approach often leads to better outcomes by addressing the unique needs of young people and their families. They also highlighted the importance of empowering parents and addressing family dynamics to ensure sustained support for CYP during recovery. However, systemic demands often compromise the quality of care in both models, leading to a reliance on quick fixes like medication instead of holistic and relational approaches. These insights reinforce the need for more adaptable, relationship-focused mental health services to support young people and their families effectively.

### Main analysis

The analysis revealed three main themes related to the in-depth experiences of healthcare professionals delivering services in the ICCS and TAU settings. The themes include “Relational Ecologies: barriers and enablers to engagement”, “Parental role in the ecosystem: enskilling parents”, “The web of trust in the relationship-building process. Participants were asked about their experience of supporting young people within their services, including some explicit references to the barriers and facilitators related to delivering their services to CYP. However, the data revealed how barriers and enablers were organised thematically around issues of relationality.

Relational Ecologies: barriers and enablers to engagement.

All HCPs characterised their experiences of working with CYPs as interactions that were part of a broader relational ecosystem. The term ecosystem here refers to the comprehensive network of therapeutic relationships and interactions present between stakeholders, including healthcare professionals, peers, and family members. Within the context of our study, there are numerous interconnected and interdependent entities present across the different services within CAMHS. According to the accounts provided by HCPs, these interdependencies among the various entities (HCPs, peers, family/guardian, and other agencies) have been identified as significant factors influencing young people’s engagement with support. This engagement encompasses the initial help-seeking phase and extends to the CYPs ongoing involvement with the services offered. A key relational form influencing service engagement was reported to be the family network, specifically familial expectations and anxieties.

*You often have to tolerate lots of that reaction, the disappointment, the distress and you quite often get told you are not doing a good job, not doing enough, being neglectful (HCP Inpatient).*

*For example, somebody that’s being admitted to a ward, we’re still involved and we’ll meet with families, one on one, without the young person, we’ll meet with the family and get their perspective and relay what we can do to navigate the issues that they’ve got so that they feel more comfortable (HCP ICCS).*

The critical role of trust between HCPs and families in delivering effective mental health care was emphasised. HCPs highlight reflective practice, showcasing their awareness of the need to ensure parents feel understood and confident in the care provided for their child. This self-awareness indicates that trust is not assumed but actively cultivated through thoughtful communication and consistent engagement. Without such trust, families are less likely to feel reassured or collaborate effectively with providers.

Additionally, as described above, HCPs frequently encounter disappointment and distress from families. The emotional toll of addressing these reactions underscores the intense relational labour required in mental health care. Parents’ dissatisfaction often stems from unmet expectations or long-standing frustrations with the healthcare system.

There is a ripple effect on how these feelings of disappointment, fuelled by prolonged waiting times, can extend to the CYP, potentially impacting their engagement with services. This dynamic reveals the cascading effect of systemic inefficiencies on relational trust and therapeutic outcomes.

*Parents are often coming from that place of disappointment with the service considering the length of time they have spent on the waiting list before getting some treatment. The disappointment often transmits to their wards and how ready they are to engage with the service (HCP Inpatient).*

Past negative experiences with mental health services are identified as significant barriers to family engagement, as noted in the quote by (HCP Inpatient) above. Even when HCPs offer a more collaborative, tailored approach, families may remain sceptical, expecting more of the same inadequate care. This scepticism can hinder the establishment of trust and delay meaningful engagement, requiring HCPs to invest additional time and effort in rebuilding relationships.

Whilst YPs are then engaged with the services, there are descriptions of the expectations of the relationships (formal and informal) that they have with the HCPs within the services. HCPs emphasise the role of relationships and trust in effective service delivery.

*We take our time to explain to the young people that we cannot force them to do anything and that our purpose is to try and put them in a better place mentally from where they are, we say to them the reason why we can’t force you to do anything is because they’re difficult to engage and if they feel they’re being pressured in any shape or form they’re just going to back off even more (HCP ICCS).*

The importance of a non-coercive approach when working with young people who are often difficult to engage was discussed, explaining to CYP that they are not being forced into treatment, as any perceived pressure could lead to disengagement. This reflects an understanding of the autonomy and agency of young people, which is critical for fostering trust. By adopting a patient-centred strategy, the HCP prioritises creating a safe, judgment-free environment where YP feel empowered rather than controlled. This aligns with trauma-informed care principles, which advocate minimising power imbalances and respecting the client’s pace [32].

Additionally, HCPs highlight the quality of relationships between young people, their families, and service providers is directly tied to positive outcomes. By emphasising “really strong relationships,” the HCP connects engagement and trust-building with better mental health progress. This insight reflects the relational nature of ICCS, where the human connection between the client and the care team is foundational for effective intervention.

*If I’m honest with you, that’s where the really good work is done, and we tend to find that outcomes improve when we have really strong relationships between the young person, the family, and those involved in their care (HCP ICCS).*

*And with ICCS, because it’s just the one clinician that’s the main contact you can develop a better relationship and hopefully have more influence (HCP ICCS).*

*I think relationships are important because especially if you’re in a crisis situation, you want to be working with someone that you feel understands you, that you don’t have to give your whole life story to again and, also they can see the progression (HCP ICCS).*

In addition, there were discussions around the role of peer relationships developed during their engagement with services. HCPs recognise that while peers can serve as a source of emotional support, they may also inadvertently perpetuate harmful behaviours, such as self-harm, through social learning or normalisation [33]. This dynamic reflects the vulnerability of adolescents who often turn to peers for help but lack the guidance of healthcare professionals equipped to address these challenges effectively.

*“So, some young people only speak with peers and they end up learning behaviours that cause harm as well, sometimes we have groups of  ...,  ... in person and you learn that they have a group of friends and a few of their friends’ self-harm, so they learn the way that they find helpful to cope with the symptoms (HCP ICCS)”.*

*“Sometimes the tricky thing is that some young people confide in their peers and that’s not always helpful because to tell another young person a secret or get support from another young peer who is also maybe struggling in adolescence is very difficult because obviously, they’re not able to support them as much as what they might need (HCP Core CAMHS)”.*

According to the data presented above, there appears to be a risk of maladaptive coping mechanisms spreading within peer groups, particularly among CYP who may share similar struggles. Discussions reveal how peer dynamics can influence behaviour in ways that hinder recovery, as adolescents may adopt unhealthy strategies observed in others. Moreover, the limitations of peer support were highlighted, pointing out that young people who are themselves struggling may lack the emotional resources or maturity to provide adequate help. This can lead to a cycle of unmet needs and reinforce feelings of isolation or distress. This suggests the importance of fostering safe, supportive environments where professional guidance complements peer interactions, helping young people develop healthier coping mechanisms and break cycles of harm within their social networks.

### Parental role in the ecosystem: Enskilling parents

This section focuses more on the role of parents and families and their contribution to facilitating engagement with services. This includes parents acting as an avenue for help-seeking for mental health concerns or determining how well young people engage with services. More specifically, HCPs acknowledged the need to upskill parents to support the CYP in their care more efficiently. Harden [34] highlighted that HCPs often undermine parents’ confidence as parents are often accused by HCPs of overacting or being overly dramatic or anxious about their child’s health concerns. Instead of recognising and validating parents’ worries, HCPs may label concerns in ways that lead to parents feeling belittled or dismissed, potentially undermining their ability to trust their instincts and seek further medical advice when necessary. As a result, the relationship between parents and healthcare providers may become strained, which can ultimately affect the quality of care and support that the child receives.

*“You have to try to always think of the young person’s best interests and balancing how the parents come into that, and I agree, sometimes it might be that they are not on the same page, so it’s trying to learn how they can be in the same place, but not in a family therapy kind of way because we don’t do that in an outreach thing, just trying to see how the parents can understand their needs a bit more, and trying to see how both sides can communicate better (HCP ICCS)”.*

While the CYP’s interests are considered a priority, the entire relational ecosystem, requires careful management in order for the intervention to succeed. This includes managing the knowledge and skills parents bring to the service and ensuring these are developed and parents empowered:

*“So, it’s skilling up the parents as well and developing an understanding and formalising the difficulties (HCP ICCS)”.*

*“Parents will be given psychoeducation about mental health illness so that they feel more comfortable speaking to the young people about that. (HCP Urgent care team)”*

Upskilling parents with the skills that empower them to take an active role in the management of their child’s recovery process seems vital.

*“We sometimes find that families will say my young person wants to be going out, can I let them, and we say you are the parent, so give them back the power, because when someone is in crisis we, as professionals, do go in and we do take a lot of the power away from parents and we can become quite restrictive initially for safety reasons, but then we have to bear in mind that it’s the family that’s living with this young person and we have to empower them to look after that young person. (HCP ICCS)”*

*“I think with education we need to help families recognise that actually mental health struggles are multi-determined and they don’t just come from one place, and I suppose one of my fears is that with labels, once the labels start to get attached, some families, not because they’re trying to blame one thing, but I think it’s because they just don’t understand all the different factors that have an influence on young people’s or on anybody’s mental health, so I think we need a more open education type system, a non-blaming education system around mental health and how everybody can influence positive mental health by the way we interact. (HCP ICCS)”*

HCPs highlighted the importance of empowering parents to take charge of their children’s well-being, particularly during times of crisis. They acknowledged professionals’ role in taking control for safety reasons but highlighted the need to empower families to ultimately care for their young person in the longer term, involving them right from the start. They also touched on the complexity of mental health struggles and the potential drawbacks of attaching labels to them. This suggests the need for a more comprehensive and non-blaming education system that alerts families to the various factors influencing mental health and how everyone can contribute to positive mental well-being through positive relationships. Together, the ICCS philosophy of relational care is highlighted here, which seems to prioritise families as central actors in a young person’s mental health journey. Empowering families requires healthcare professionals to step back and equip parents with knowledge and confidence. This approach also reflects an acknowledgement of systemic power dynamics in healthcare, where professionals must navigate between being agents of immediate action and facilitators of long-term family-led care.

To the role of parents in the care and support of young people (YP) with mental health concerns is considered by HCPs to be crucial. Beyond educating and empowering parents/guardians, HCPs emphasise the role of developing a relationship with family, especially in ICCS, to improve engagement levels.

*I think it’s always been relatively straightforward, we will be working with children that don’t want us there, but we will be working with parents that do, and so our ability to get them on board is relatively straightforward (HCP ICCS).*

*even the parents have to do a lot of parent work where the parent would call me every day and say we’ll have a chat about what’s going on, and I think they found that a bit comforting, and obviously they needed that, but I guess it’s not attainable for a long period of time, so we had to work on independence quite a few times (HCP ICCS).*

*Often having conversations with the parents, knowing that they’re in a position where they probably do feel like they’re failing and that they are responsible (HCP ICCS).*

HCPs recognise the initial control taken by professionals for safety reasons but stress the importance of ultimately empowering families to care for their YP. Furthermore, there is a call to integrate the family’s role as a perduring support system with the aim of creating a collaborative framework that promotes resilience for both the YP and their caregiver.

### The web of trust in the relationship-building process

HCPs play a crucial role in the relational ecosystem, which is the central theme discussed here. The length of time required to build meaningful therapeutic relationships and their intersection with gaining the trust of parents and young people were mainly discussed by the HCPs. Whilst HCPs appeared to be time pressured to engage with YP in the various services, gaining trust seems vital to the dynamic relationship between HCPs and YP and well worth the temporal investment [35].

*“Lots of our service delivery is about the relationship and how sometimes you need to spend quite a long time than you do in other services building up the relationship, and sometimes it’s  ..., sometimes it’s coffee or bubble tea, or sometimes playing a game with them during lunchtime when they are in the day service and that’s part of the relationship building up. (HCP ICCS)”*

*“I think in our service, we are actually meant to be a short-term service of 12 weeks and often we will extend beyond that because of the lack of engagement and so in terms of 12 weeks I would say that isn’t enough but we often and do extend beyond that. I have worked with someone for nearly a year before so there is flexibility, we have that luxury of flexibility in that we don’t have a waiting list so we can do that. (HCP Inpatient)”*

*“The last thing they want to do is go into a clinic and say here’s all my stuff. And to do that takes time, it takes the person’s trust, it takes the ability for them to realise that I’m not going to get in trouble for this stuff and actually, I might get some help for it, and then tailoring that help as much as you can for whatever is going on for them. (HCP Core CAMHS)”*

*“Then I think in our work it’s really not having an agenda, so at the moment we’re very lucky, we don’t have targets we have to meet, there’s no KPIs, there’s none of this that we are stuck to in terms of stuff we have to get done, we are getting them done, but we’re getting them done in our own way which I think is another really helpful thing (HCP ICCS)”.*

Based on the above extracts, HCPs emphasise the significance of building strong relationships in service delivery. They highlight the importance of spending time and engaging in various informal relational activities, such as having coffee or playing games, to develop a strong rapport with clients, prioritising personalised and relational aspects of service delivery.

In addition, the flexibility of service duration was also described as crucial, extending beyond the standard 12 weeks to accommodate the need for continued engagement and support. This call for flexibility recognises the inadequacy of a fixed time frame in addressing clients’ needs. Time, trust, and tailored support are required for clients to open up and seek help. The need for flexibility in approach underscores the need for a non-clinical, more informal, trusting environment and the importance of personalised assistance to address clients’ unique circumstances.

*“There are so many people, so many different parts. Somewhere between three and five percent of the people come from CAMHS, I think that’s the amount that we get per year, so we are only dealing with a very, very small percentage of people so that means that so many children who go into CAHMS have their therapies and come out fine; that’s a lot. When I’m working with someone, I am working with horrendous stuff so home treatment is when working in a clinic hasn’t worked, talking therapies haven’t worked, DBT or whatever therapies haven’t worked and when we don’t see things working in an environment like a clinic perhaps we can give them home treatment and come out and work with you there. So, we hit that 3% of people with a comfortable place to work, a more personable ‘let’s have a look at this, let’s have a look at that’ mentality and then be able to draw in the therapies, sometimes without the kids even realising that they’re doing that (HCP ICCS)”.*

*“The young person and the families, in general, they’re not going to respond to any treatment if they don’t engage, they don’t trust. So that is pretty key: it’s very important for them to trust us. (HCP ICCS)”*

*“I think the relationships, they have to trust you and you have to trust them as well and that’s the basic relationship that you have to have with them and then develop the engagement. It may be that I go to them, I go to their house the first day and I don’t do a medical assessment or anything, I just go there to engage with them and I may have to go a few times to be able to engage with them because they may not trust you – who is this person coming to my house who I’ve never met? So, that is why it is really important that we try to develop that engagement as soon as possible to be able to provide the service. (HCP ICCS)”*

These quotes emphasise the importance of building trust and establishing a strong relationship between HCPs and CYPs and their families. Particularly in the community setting (ICCS), HCP stressed that without trust and engagement, any treatment or service provided would not be effective. They highlight the need to invest time in developing trust, even if it means making multiple visits without immediately focusing on any formal medical assessments. Building trust and engagement was seen as the foundation for successful healthcare outcomes

and the flexibility built into the approach was seen as a key element in strengthening the cord of trust:

*“Some people work more flexibly; some people work more proactively, and other people might not. It’s a clinical style: different people have a different approach (HCP Core CAMHS)”.*

*“That they feel listened to and understood, they don’t feel you are trying just to go through a checklist of items because they are all so used to that because usually the young people that come to us are not new in CAMHS (HCP ICCS)”.*

*“Some might like a certain approach; others might not like that: so, a professional being able to adapt to that (HCP ICCS)”.*

*“I never feel old, so when I’ve got someone in the car or if I’m in someone’s house or if I’ve taken them to a café I chat about what they want to chat about to start off with, I ask them from their point of view and if I don’t understand something or don’t know something I will say I don’t understand that, what is the attraction to that, why do people like that band. And if they’re talking about something like Love Island I can turn round and say from my point of view materialism, oh that’s pretty bad, but you lot seem to like it, why is that, and they will be able to give you the answers, and so you can work out where their moral values are, you can work out almost what lessons they’ve missed (HCP ICCS).”*

*“I once built a rapport with a boy who had autism and refused to speak to every other professional from our team when he was assessed previously, and I used 15 minutes of my appointment speaking about a Michelin star restaurant he went to in London and about food and how different flavours can make a meal better etc, so that is how I developed a rapport (HCP CAHMS Rapid response team)”.*

The data extracts offer insights into the diverse and personalised approaches employed by HCPs. They underscore the significance of flexibility, active listening, adaptability, and rapport-building in clinical practice. The emphasis on recognising individual differences, engaging in empathetic dialogue, and tailoring interactions to meet the unique needs of individuals highlights the personalised and human-centric nature of mental health care.

*In inpatient settings, they have got loads of different people to talk to and people offer different things to them and they are generally quite good at going to different people for different things, based on what they get from them but I think sometimes it is a bit confusing to them if they feel they have heard different things from different people, there is the potential for splitting or whatever. I think that even though I feel like everyone can have an input in decision making, I think often the young people feel that everything goes to the consultant and she’s the only person who has the power really to do anything (HCP inpatient).*

The above extract signals an acknowledgement of the variety of professionals available to service users, which can be beneficial as individuals seek different types of support from various team members particularly in inpatient settings. This diversity is important because it allows patients to address specific needs by reaching out to those best suited to assist them. However, this also points to a significant issue regarding the potential for confusion when patients receive differing information from multiple sources. This inconsistency can lead to feelings of fragmentation or “splitting,” where patients may struggle to reconcile conflicting messages from different HCPs.

Additionally, other members of the clinical team could take a more proactive role in decision-making rather than deferring to the lead consultant. Empowering all members of the team to make decisions, like in the ICCS model, could enhance collaboration and patient autonomy, allowing for a more inclusive approach to care. This collaborative decision-making model can diminish confusion for the patients by providing them with consistent messaging and support from a cohesive team, improving their overall experience and satisfaction with the care process.

Although the themes were developed across the combined dataset, healthcare professionals described some differences in how relational care was enacted within ICCS and TAU. Table 3 provides a comparative synthesis of aspects particularly emphasised within each service model, alongside experiences consistently reported across both services.

**Table 3:** Comparative synthesis of healthcare professionals’ experiences across Intensive Community Care Services (ICCS) and Treatment as Usual (TAU).

Theme	Treatment as Usual (TAU)	Intensive Community Care Services (ICCS)	Shared experiences across both services
**Relational Ecologies**	Therapeutic relationships were recognised as important but were often constrained by limited appointment availability, service thresholds and fragmented pathways. Professionals described difficulties sustaining engagement with young people and families within existing service structures.	Professionals described greater flexibility to engage young people in community settings, spend more time building relationships and adapt care to individual and family circumstances. Family engagement was viewed as a central component of intervention delivery.	Across both services, participants considered strong therapeutic relationships fundamental to successful engagement. Family dynamics, peer influences and the wider social environment were consistently viewed as shaping young people’s recovery and service engagement.
**Parental Role in the Ecosystem**	Parents were recognised as important partners in care, although opportunities for ongoing collaboration and psychoeducation were sometimes limited by service capacity and episodic contact.	Parents were actively supported through psychoeducation, collaborative decision-making and practical strategies that increased confidence in managing their child’s mental health. Professionals described working alongside families rather than solely treating the young person.	Participants across both services viewed parents as essential contributors to recovery. Supporting parental understanding, confidence and involvement was regarded as improving treatment engagement and long-term outcomes.
**Web of Trust**	Trust was developed primarily through consistency, empathy and professional communication, but was often challenged by waiting times, staff changes and transitions between services.	Trust was strengthened through intensive, flexible and sustained contact, including home visits and community-based engagement. Professionals described continuity and responsiveness as facilitating deeper therapeutic relationships.	Healthcare professionals across both services identified trust as the foundation of effective care. Continuity, authenticity, clear communication and respect were viewed as essential for engaging young people experiencing complex mental health difficulties.

## Discussion

This study explored healthcare professionals’ experiences of delivering ICCS and TAU within Child and Adolescent Mental Health Services (CAMHS), with particular attention to the relational, organisational and contextual factors that shape engagement, care delivery and recovery. Although many of the themes were evident across both ICCS and TAU services, healthcare professionals working within ICCS more frequently described opportunities for flexible engagement, intensive family involvement and continuity of care. In contrast, professionals working across both service models consistently identified therapeutic relationships, trust-building and organisational pressures as fundamental influences on care delivery. These findings suggest that while relational practice is valued across CAMHS services, ICCS may provide organisational conditions that better support its implementation.

The findings reveal a nuanced balance of barriers and enablers that influence effective service delivery. ICCS staff emphasised key enablers such as the flexibility to deliver care in homes and schools, strong family engagement, and a team-based approach that allowed holistic, context-driven interventions. Staff described how providing treatment in a young person’s everyday environment helped maintain normal routines and family connections, aligning with known advantages of community-centred care (e.g., preventing isolation from school/family and enabling holistic assessment) [2]. They felt this approach fostered trust and sustained improvements, echoing published reports that ICCS settings can increase the likelihood of sustainable change in outcomes. Notably, HCPs gave examples of working creatively with families, for instance, scheduling sessions around school or meeting in informal settings, which they saw as critical facilitators of engagement. These accounts underscore how ICCS leverages the home and community context as a therapeutic asset, consistent with literature highlighting that community-based treatment can reintegrate young people early and avoid the disruptions of inpatient admission [2]. In turn, TAU staff often working in traditional inpatient or clinic settings described different challenges. They noted barriers like the fragmentation of care when transitioning patients back home and the limit of short inpatient stays in addressing complex family dynamics. Some TAU practitioners expressed frustration that, without an intensive community option, young people “bounced” between hospital and home with insufficient support. This perspective reinforces why ICCS models were developed to fill the gaps left by standard services [2]. Indeed, our findings illustrate that many barriers in TAU (e.g., abrupt discharge, loss of continuity) are the very issues ICCS is designed to mitigate by providing extended, home-based support.

Despite the strengths of ICCS, HCPS in both TAU and ICCS candidly discussed significant barriers to effective delivery. Resource and capacity constraints were a prominent theme. ICCS teams often carried small caseloads by design, yet staff still reported high workload intensity given the complexity of cases and the 24/7 nature of support. Some ICCS interviewees described the emotional toll of managing risk in the community; responding to crises at odd hours was seen as necessary but taxing, raising concerns about burnout. Likewise, TAU staff highlighted chronic understaffing and bed shortages that hampered their ability to provide high-quality care – an issue consistent with wider systemic problems noted in prior analyses (e.g., stakeholder reports of an overstretched CAMHS system). Another barrier was inter-agency collaboration. ICCS staff sometimes struggled to coordinate with schools, social services, or inpatient units, especially when roles were unclear or when colleagues in traditional settings were sceptical of the community approach. This finding echo previous observations that ICCS and similar teams often operate in isolation without shared protocols, underscoring calls to better integrate these services into the broader system [36]. Safety and risk management were identified as a further challenge. TAU staff were concerned about certain high-risk CYPs being managed at home, while ICCS staff acknowledged feeling “on their own” when handling severe crises in the field. Importantly, HCPs described how strong team support and supervision were essential enablers to overcome these stresses. For example, regular team debriefs after crises and a culture of collective problem-solving helped staff feel more confident and supported. This points to the critical role of team cohesion and training as enabling factors in both models. These qualitative insights build on and enrich the existing research on intensive community care. Previous studies have mainly quantified outcomes, showing that intensive community treatments can reduce hospital use and improve patient satisfaction relative to usual care [15]. Our findings help explain how those outcomes may be achieved. For instance, staff attributed reduced admissions to proactive work in the community, “keeping the young person safely at home,” which aligns with evidence that ICCS is associated with fewer and shorter hospital stays. They also observed better engagement and trust from families, echoing reports of greater patient/family satisfaction under community-based models. By detailing the mechanisms (e.g., building rapport in familiar environments, addressing problems in real-time at home), this study provides context for the positive quantitative outcomes on the effectiveness of home-treatment care [15,26]. Our findings support the view that intensive community care can be effective and person-centred, but its success depends on implementation factors. When those factors are favourable (adequate staffing, inter-agency support, skilled clinicians), ICCS fulfils its promise of comprehensive, home-based care. When they are not, even a well-intended ICCS can fall short of a reality that highlights why ongoing evaluation and refinement of these services are critical.

Taken together, the overarching themes identified suggest both shared and service-specific emphases in healthcare professionals’ experiences. Across both ICCS and TAU, participants consistently described therapeutic relationships, trust, and meaningful family involvement as fundamental to engaging children and young people with complex mental health needs. Professionals working across both service models recognised that recovery extended beyond symptom reduction and depended on understanding the wider relational and social contexts in which young people lived. However, participants working within ICCS more frequently emphasised the organisational conditions that enabled these relational approaches to be enacted in practice. In particular, they described greater flexibility in the timing and location of care, opportunities for sustained engagement through community- and home-based contacts, and the capacity to respond rapidly to changing clinical and family needs. In contrast, professionals working within TAU more commonly reflected on organisational constraints, including service pressures, limited contact time and fragmented care pathways, which could restrict opportunities to develop and maintain therapeutic relationships. These findings suggest that although the principles of relational care are valued across both service models, ICCS may provide organisational structures that better facilitate their implementation. Rather than indicating fundamentally different philosophies of care, the findings imply that the intensity, flexibility and continuity afforded by ICCS may strengthen professionals’ ability to operationalise relational practice with children, young people and their families. Future research should move beyond examining whether intensive community care is effective to understanding how relational models of care can be implemented and sustained within routine clinical practice. Longitudinal studies should evaluate the durability of outcomes for children and young people, while qualitative research involving families, carers and underserved populations would provide a more comprehensive understanding of how these models are experienced across diverse communities. In addition, implementation and process evaluations are needed to identify the organisational conditions, workforce requirements and contextual factors that support the successful delivery of ICCS at scale.

While ICCS offers organisational conditions for relational, family-centred, flexible care, scaling up requires careful planning of workforce capacity and service infrastructure. Its intensive, community-based approach with multidisciplinary collaboration needs more staffing, protected time, and ongoing organisational investment than standard outpatient care. This should align with the pressures on CAMHS, including rising demand, workforce shortages, and resource limits. Wider ICCS implementation requires workforce planning, staff training, supervision, and support to remain effective and sustainable. Although this study did not assess cost-effectiveness, future research should include economic evaluations alongside effectiveness and implementation studies. This will help determine if the investment in ICCS reduces hospital admissions, crisis cases, and long-term service use, guiding broader adoption within CAMHS [37].

### Strengths and Limitations

This study has several strengths. By capturing first-hand accounts from staff across three distinct service models, it provides a rich, comparative perspective rarely seen in the literature. The use of thematic decomposition enabled a thorough analysis of the meanings associated with relational practices. The in-depth thematic analysis yielded insights into the day-to-day facilitators and obstacles in service delivery, offering depth and context to complement the Trial element. Another strength was the inclusion of participants from multiple professional backgrounds (e.g., nurses, social workers, psychiatrists) across ICCS and TAU. This diversity increased the breadth of perspectives, enhancing the credibility of the themes by showing they were not isolated to one role or site. We also explicitly examined two contrasting models (ICCS vs. TAU), which strengthens the analysis by allowing us to distinguish which challenges are model-specific and which are more general in children’s mental health services.

However, the study only captured staff perspectives. It did not include direct accounts from young people or families, which limited the ability to triangulate the findings fully. However, clear points of comparison can be made with a separate article reporting data with young people from this study [38].

Furthermore, interviews were conducted within the framework of an RCT, which may not entirely reflect real-world service delivery outside of research settings.

### Implications for Practice

These findings suggest that relational models such as ICCS should be further integrated into mainstream CAMHS delivery. Specifically, the development of ICCS should focus on building an effective support system for young people. It is essential to prioritise flexible, individualised approaches that accommodate their preferences for time, location, and method of engagement. Strengthening staff training in trauma-informed, non-coercive, and culturally sensitive care practices is crucial for providing the best possible support. Additionally, actively involving parents and carers through psychoeducation will empower them as partners in the recovery process, further enhancing the effectiveness of interventions.

To simplify access to care, developing strategies that address systemic barriers is vital, particularly by reducing waiting times for therapy and equipping young people with clearer and more transparent navigation tools for services. Managing peer influences is also important; implementing professionally supported peer programs can help prevent the spread of maladaptive behaviours among youth. Previous community mental health initiatives have demonstrated that digital platforms can improve engagement and access to care, particularly when integrated within broader community-based service models [16]. The experiences of healthcare professionals in the present study suggest that these technologies may further enhance the relational principles that characterised ICCS by supporting communication between appointments, facilitating continuity of care and strengthening family involvement. Rather than replacing therapeutic interactions, digital tools have the potential to extend relationship-based care beyond the clinic through flexible, responsive contact and coordinated multidisciplinary communication.

Moreover, it is crucial to protect staff from relational fatigue by ensuring manageable caseloads and providing reflective supervision spaces that focus on the relational aspects of their work. Building these relational infrastructures necessitates a concurrent commitment to addressing broader systemic constraints, such as workforce shortages and rigid care pathways, to ensure long-term sustainability and effectiveness of the support provided. Delivering intensive relational care demands significant emotional labour. Professionals face traumatic or stressful situations linked to mental health issues, including burnout, depression, and sickness absence [39]. Emotional labour, often involving “surface acting,” depletes psychological resources and leads to exhaustion and disengagement [40]. The Job Demand–Resources model shows that high demands, like heavy caseloads, cause strain when resources are lacking, resulting in burnout characterised by exhaustion and cynicism that impairs performance and causes turnover [41]. To sustain relational models like ICCS, organisations must support workforce wellbeing. Clinical supervision and reflective practice can reduce burnout and turnover [42]. Effective supervisors provide regular supervision, discuss demanding cases, offer feedback, and create trust-based environments, which enhance the benefits of deep acting and lessen the negative effects of surface acting on job satisfaction and burnout.

More broadly, these findings suggest that implementing relational models of care requires investment in the organisational conditions that enable them to flourish. Alongside workforce planning, services should support clinicians through regular supervision, reflective practice and training in trauma-informed and family-centred care. Strengthening multidisciplinary collaboration and embedding family psychoeducation within routine service delivery may further enhance engagement while maintaining the flexibility that professionals identified as a defining feature of effective community-based care.

## Conclusion

This study provides compelling evidence that relational care, underpinned by trust, flexibility, and family engagement, is central to successful community-based mental health interventions for CYP. ICCS offers a promising model by embedding these relational principles into service delivery. However, systemic challenges, including workforce limitations and service pressures, continue to undermine these efforts. Future service models must combine relational excellence with structural reform to ensure that all young people accessing mental health services experience care that is personalised, empowering, and effective. Further research exploring young people’s direct experiences and the scalability of relational care models will be critical for informing national service redesign.

## Supporting information

S1 File This is the S1 File Qualitative Interview Schedule. (PDF)
