# Supplementary material for: Relational care in community mental health: Evaluating staff experiences in Intensive Community Care Services (ICCS) vs treatment as usual
Source: PLoS One. 2026 Sep 9;21(9):e0356608. doi: 10.1371/journal.pone.0356608 (PMC13557356; doi:10.1371/journal.pone.0356608)
Supplement: Supplementary file 1 [file pone.0356608.s001.pdf]

Supporting information file S1 Qualitative Interview Schedule

Notes to the interviewer will be written in italics. Please note the following:

- *This interview consists of five sections and should last an hour in total*
- *This is a rough guide, so please don't cut an avenue short if you feel it will lead to valuable information. Ensure that you have longer than an hour available if needs be.*
- *Please be mindful that you are not overtly checking the time, as this can result in participant disengagement.*

Icebreaker

Start by simply Introducing yourself, reminding them very briefly what the study Is about and then begin by asking them about themselves – what they do, what they like doing in their spare time, and what they like about their job. How long have they been working there? The rapport stage is important, as it sets the tone for the interview.

- *The interview will begin with participants introducing themselves and stating if they work in inpatient services or the ICCS services they have been assigned.*
- *Ask participants to explain their role within the service model, either inpatient or ICCS. Make sure they clarify how their role fits within the entire service model.*

1. **What would you say are the most common mental health issues among young people presenting to acute CAMHS services?**

Prompts:

- In their words, ask them to describe what they think are the common issues that young people present with and ask them to describe what they think causes these common issues (Presentation, referrals, engagement with services)
- What do you think are the common causes of mental health difficulties amongst young people? Ask them to describe common mental health difficulties based on their experience of managing them.
- Who do they think children and adolescents turn to when their mental health becomes an issue?
- In their view, which service offers the most beneficial care model? Ask them to explain their answer in detail, with examples. Please ask them to situate their answers in terms of the client group – i.e., young people and ask them to describe whether the service fits the developmental stage of the patients.
- What information is provided to children and their parents about mental health services?
- Do YP and their families understand what the purpose of the service is and what it sets out to do? What support do they have for that view?

2. **How would you describe the service you were assigned to?**

   Prompts

   - What do you think are the important drivers of the service? What is it there to achieve?
   - What is the main treatment model being used? Depending on what they say (medical, systemic etc.), ask them to provide detail on how they think this works and what evidence there is to support it.
   - What makes it different to other services you are familiar with?
   - What is the environment like to work in? Ask them to describe how they think the environment helps support the aims of the service or whether there are problems with the environment itself. Ask for examples.
   - What is the environment like for the service users? Same prompts.
   - Do you think the service is effective? Ask for detail here as we want to drill down into the actual experiences of working in the service, including the barriers encountered. So, the why, how, and what are effective questions.
   - Is this service the best place for YP in crisis? Ask them to give several reasons for their answers. Ask them what they think might be a better way forward and focus on what they would propose as an alternative.

3. **How would you characterise the service you were allocated to (Inpatient/ICCS)? – What is its aim, and what is involved?**

   - Without giving out names and details of participants, can you give us an example of where the service was delivered?
   - Do you think children and their parents understand the service and what it involves?
   - Thinking about the role and involvement of parents/guardians - Is their engagement important to the success of the service? Or can the service be delivered independently without their involvement?
   - Were there any complications in trying to explain the service to them?
   - How do you think young people have engaged with the service? From your perspective, why do you think it has/hasn't worked for them? Try to encourage them to provide examples.
   - Are there any dilemmas you have encountered within the service regarding keeping YP safe? Here we are asking them about risk, so how does the service deal with risk issues and have they had any problems with risk etc.?

4. **How would you describe your relationship with service users in this service?** –
   What do you think is the role of relationships in delivering the intervention? Ask them to be very specific here -what kinds of relationships are possible, are beneficial and which ones are not.
   - Do you think there is sufficient space within the service to develop meaningful relationships with SUs? Again, ask how, why and what. Have they encountered any barriers, for example? Is there enough time and space – is the environment set up to facilitate this?

- Encourage them to provide details.
- What makes a good relationship between staff and SUs?
- Does your current services allow you some space and the means to develop this good relationship? Encourage them to provide some details.
- How do you ensure a balance between your relationship with the service user (child) and their parents/guardian? Are there any difficulties you have come up against in trying to maintain this balance? Could you provide an example of how this balance can work or not?

5. Once the young person is ready, how would say you prepare them for their recovery? What sort of things do you do?

   - How would you describe recovery within the service model you are currently allocated to? What indicators are there to suggest the YP has recovered? What makes for a good recovery?
   - What are the things you put in place to ensure the YP understands what is involved in their recovery? Do you provide support in terms of how to recognise difficulties that may return? What does this support involve?
   - How much do you think having more than one team involved in their care influences their recovery? In a positive way? Not so positive? Try to encourage them to talk about any arising complexities from working across teams. Any difficulties they have encountered?
