# Supplementary material for: Relational care in community mental health: Evaluating staff experiences in Intensive Community Care Services (ICCS) vs treatment as usual
Source: PLoS One. 2026 Sep 9;21(9):e0356608. doi: 10.1371/journal.pone.0356608 (PMC13557356; doi:10.1371/journal.pone.0356608)
Supplement: Supplementary file 2 [file pone.0356608.s002.pdf]

PLOS One

Relational Care in Community Mental Health: Evaluating Staff Experiences in Intensive Community Care Services (ICCS) vs Treatment as Usual.

--Manuscript Draft--

**Manuscript Number:**	PONE-D-26-13555
**Article Type:**	Research Article
**Full Title:**	Relational Care in Community Mental Health: Evaluating Staff Experiences in Intensive Community Care Services (ICCS) vs Treatment as Usual.
**Short Title:**	Relational Care in Community Mental Health: Staff Experiences in ICCS vs Usual Care.
**Corresponding Author:**	Opeyemi Atanda<br>London South Bank University School of Applied Sciences<br>London, UNITED KINGDOM OF GREAT BRITAIN AND NORTHERN IRELAND
**Keywords:**	Adolescent mental Health; therapeutic alliance; community mental health services; inpatient psychiatric care; service engagement; Recovery; relational dynamics; qualitative research.
**Abstract:**	Background: The quality of healthcare delivery relies heavily on building strong relationships between healthcare providers (HCPs) and clients. This study presents the results of a qualitative interview study with healthcare professionals. This study formed part of a process evaluation for a Randomised Controlled Trial (RCT) examining the effectiveness of Intensive Community Care Service (ICCS) vs Treatment as Usual (TAU; inpatient or core community CAMHS).<br>Methods: This paper is based on a process evaluation examining how healthcare professionals experience both services, specifically focusing on their perceptions of young people's service engagement and recovery journey. Thirty-five semi-structured interviews were conducted with staff across various services, including 19 from ICCS and 16 other TAU services. A thematic decomposition analysis was conducted on the data, and specific themes relevant to staff experiences of young people's engagement with services and overall recovery were examined in-depth.<br>Results: Three main themes were observed in the HCP data (1. Relational Ecologies: barriers and enablers to engagement, 2. flexibility of approach amidst systemic pressures and 3. the web of trust in the relationship-building process). HCPs highlighted the necessity of developing trust and rapport through non-clinical engagement strategies, such as informal visits and personalised interactions. HCPs emphasised that without trust, treatment effectiveness diminishes, necessitating a tailored approach rather than a one-size-fits-all model. The flexibility in duration of treatment and methods of engagement were noted as crucial in accommodating individual client needs and fostering an open, trusting environment necessary for long-term recovery.<br>Conclusion: The findings highlight the vital importance of relational care models, especially ICCS, in addressing the complex needs of CYP. Flexible, family-centred approaches improve trust, engagement, and long-term recovery outcomes. Recommendations include tackling systemic barriers and expanding relational care models within CAMHS to meet increasing mental health demands effectively. Further research should investigate scalable strategies for integrating these insights into wider mental health service frameworks.
**Order of Authors:**	Opeyemi Atanda
	Paula Reavey
	Ben Wong
	Ebony Baker
	Jessica Collier
	Thilipan Thaventhiran
	Veronika Dobler

Powered by Editorial Manager® and ProduXion Manager® from Aries Systems Corporation

	Ruth Woolhouse
	Toby Zundel
	Joe Clacey
	Jovanka Tolmac
	Izabela Pilecka
	Leon Wehncke
	Tauseef Mehdi
	Rhys Bevan-Jones
	Dennis Ougrin
**Additional Information:**	
**Question**	**Response**
**Financial Disclosure**<br><br>Enter a financial disclosure statement that describes the sources of funding for the work included in this submission. Review the submission guidelines for detailed requirements. View published research articles from *PLOS ONE* for specific examples.<br><br>This statement is required for submission and **will appear in the published article** if the submission is accepted. Please make sure it is accurate.<br><br>**Funded studies**<br>Enter a statement with the following details:<br>• Initials of the authors who received each award<br>• Grant numbers awarded to each author<br>• The full name of each funder<br>• URL of each funder website<br>• Did the sponsors or funders play any role in the study design, data collection and analysis, decision to publish, or preparation of the manuscript?<br><br>Did you receive funding for this work?	Yes
Please add funding details.<br>as follow-up to "**Financial Disclosure**<br><br>Enter a financial disclosure statement that describes the sources of funding for the work included in this submission. Review	This study was part of a randomised controlled trial, funded by the NIHR HTA Programme (Ref: NIHR127408). The funder plays no role in the trial design, delivery, and interpretation of data, the writing of the report, and the decision to submit the report for publication.

Powered by Editorial Manager® and ProduXion Manager® from Aries Systems Corporation

the submission guidelines for detailed requirements. View published research articles from *PLOS ONE* for specific examples.<br><br>This statement is required for submission and **will appear in the published article** if the submission is accepted. Please make sure it is accurate.<br><br>**Funded studies**<br>Enter a statement with the following details:<br>• Initials of the authors who received each award<br>• Grant numbers awarded to each author<br>• The full name of each funder<br>• URL of each funder website<br>• Did the sponsors or funders play any role in the study design, data collection and analysis, decision to publish, or preparation of the manuscript?<br><br>Did you receive funding for this work?"	
Please select the country of your main research funder (please select carefully as in some cases this is used in fee calculation).<br>as follow-up to "**Financial Disclosure**<br><br>Enter a financial disclosure statement that describes the sources of funding for the work included in this submission. Review the submission guidelines for detailed requirements. View published research articles from *PLOS ONE* for specific examples.<br><br>This statement is required for submission and **will appear in the published article** if the submission is accepted. Please make sure it is accurate.	UNITED KINGDOM - GB

**Funded studies**<br>Enter a statement with the following details:<br>• Initials of the authors who received each award<br>• Grant numbers awarded to each author<br>• The full name of each funder<br>• URL of each funder website<br>• Did the sponsors or funders play any role in the study design, data collection and analysis, decision to publish, or preparation of the manuscript?<br><br>Did you receive funding for this work?"	
**Competing Interests**<br><br>Use the instructions below to enter a competing interest statement for this submission. On behalf of all authors, disclose any competing interests that could be perceived to bias this work—acknowledging all financial support and any other relevant financial or non-financial competing interests.<br><br>This statement is required for submission and **will appear in the published article** if the submission is accepted. Please make sure it is accurate and that any funding sources listed in your Funding Information later in the submission form are also declared in your Financial Disclosure statement.<br><br>View published research articles from *PLOS ONE* for specific examples.	The authors have declared that no competing interests exist.

Powered by Editorial Manager® and ProduXion Manager® from Aries Systems Corporation

**NO authors have competing interests**<br><br>Enter: *The authors have declared that no competing interests exist.*<br><br>**Authors with competing interests**<br><br>Enter competing interest details beginning with this statement:<br><br>*I have read the journal's policy and the authors of this manuscript have the following competing interests: [insert competing interests here]*<br><br>* typeset	
**Ethics Statement**<br><br>Enter an ethics statement for this submission. This statement is required if the study involved:<br><br>• Human participants<br>• Human specimens or tissue<br>• Vertebrate animals or cephalopods<br>• Vertebrate embryos or tissues<br>• Field research<br><br>Write "N/A" if the submission does not require an ethics statement.<br><br>General guidance is provided below. Consult the submission guidelines for detailed instructions. **Make sure that all information entered here is included in the Methods section of the manuscript.**	Ethical approval was obtained from the West Midlands and Black Country Research Ethics Committee and the Health Research Authority in the UK, REC reference: 20/WM/0069. The trial was conducted in compliance with the principles of the Declaration of Helsinki (1996), the principles of GCP and in accordance with all applicable regulatory requirements, including but not limited to the UK policy framework for health and social care research. The Sponsor trust granted Local Research and Development approval. Informed consent was obtained from all participants.

Powered by Editorial Manager® and ProduXion Manager® from Aries Systems Corporation

**Format for specific study types**<br><br>**Human Subject Research (involving human participants and/or tissue)**<br>• Give the name of the institutional review board or ethics committee that approved the study<br>• Include the approval number and/or a statement indicating approval of this research<br>• Indicate the form of consent obtained (written/oral) or the reason that consent was not obtained (e.g. the data were analyzed anonymously)<br><br>**Animal Research (involving vertebrate animals, embryos or tissues)**<br>• Provide the name of the Institutional Animal Care and Use Committee (IACUC) or other relevant ethics board that reviewed the study protocol, and indicate whether they approved this research or granted a formal waiver of ethical approval<br>• Include an approval number if one was obtained<br>• If the study involved *non-human primates*, add *additional details* about animal welfare and steps taken to ameliorate suffering<br>• If anesthesia, euthanasia, or any kind of animal sacrifice is part of the study, include briefly which substances and/or methods were applied<br><br>**Field Research**<br><br>Include the following details if this study involves the collection of plant, animal, or other materials from a natural setting:<br>• Field permit number<br>• Name of the institution or relevant body that granted permission	
**Data Availability**<br><br>Authors are required to make all data underlying the findings described fully available, without restriction, and from the time of publication. PLOS allows rare exceptions to address legal and ethical concerns. See the PLOS Data Policy and FAQ for detailed information.	No - some restrictions will apply

A Data Availability Statement describing where the data can be found is required at submission. Your answers to this question constitute the Data Availability Statement and **will be published in the article**, if accepted.<br><br>**Important:** Stating 'data available on request from the author' is not sufficient. If your data are only available upon request, select 'No' for the first question and explain your exceptional situation in the text box.<br><br>Do the authors confirm that all data underlying the findings described in their manuscript are fully available without restriction?	
**Describe where the data may be found in full sentences. If you are copying our sample text, replace any instances of XXX with the appropriate details.**<br><br>• If the data are **held or will be held in a public repository**, include URLs, accession numbers or DOIs. If this information will only be available after acceptance, indicate this by ticking the box below. For example: *All XXX files are available from the XXX database (accession number(s) XXX, XXX.).*<br>• If the data are all contained **within the manuscript and/or Supporting Information files**, enter the following: *All relevant data are within the manuscript and its Supporting Information files.*<br>• If neither of these applies but you are able to provide **details of access elsewhere**, with or without limitations, please do so. For example:<br><br>*Data cannot be shared publicly because of [XXX]. Data are available from the XXX Institutional Data Access / Ethics Committee (contact via XXX) for researchers who meet the criteria for access to confidential data.*<br><br>*The data underlying the results presented in the study are available from (include the name of the third party*	Data cannot be shared publicly because they contain sensitive, identifiable information relating to participants' mental health experiences and workplace contexts, and sharing these data could compromise participant confidentiality and ethical commitments. Data are available from the West Midlands and Black Country Research Ethics Committee and the Health Research Authority (UK) (contact via hra.approval@nhs.net) for researchers who meet the criteria for access to confidential data.

and contact information or URL).<br>• This text is appropriate if the data are owned by a third party and authors do not have permission to share the data.<br><br>* typeset	
Additional data availability information:	

Powered by Editorial Manager® and ProduXion Manager® from Aries Systems Corporation

Manuscript

Click here to access/download;Manuscript;Staff paper Version - submission to PLOS.docx

Relational Care in Community Mental Health: Evaluating Staff Experiences in Intensive Community Care Services (ICCS) vs Treatment as Usual.

A paper by Opeyemi Atanda*[1], Paula Reavey[1], Ben Wong[2], Ebony Baker[1], Jessica Collier[1], Thilipan Thaventhiran[3], Veronika Dobler[4], Ruth Woolhouse[2], Toby Zundel[5], Joe Clacey[6], Jovanka Tolmac[7], Leon Wehncke[8], Tauseef Mahdi[9], Rhys Bevan-Jones[10], Izabela Pilecka[11], Dennis Ougrin[3].

[1] - London South Bank University

[2] - East London NHS Foundation Trust

[3] - Queen Mary University of London

[4] - Cambridge University Hospitals NHS Foundation Trust

[5] - South London and Maudsley NHS Foundation Trust

[6] - Oxford University Hospitals NHS Trust

[7] - Central and Northwest London NHS Foundation Trust

[8] - Northeast London NHS Foundation Trust

[9] - Royal Berkshire NHS Foundation Trust

[10] - Cwm Taf Morgannwg University Health Board

[11] – Kings College London

***Corresponding author**

E-mail: atandao4@lsbu.ac.uk

(IVY trial ref: NIHR127408)

Abstract

Background: The quality of healthcare delivery relies heavily on building strong relationships between healthcare providers (HCPs) and clients. This study presents the results of a qualitative interview study with healthcare professionals. This study formed part of a process evaluation for a Randomised Controlled Trial (RCT) examining the effectiveness of Intensive Community Care Service (ICCS) vs Treatment as Usual (TAU; inpatient or core community CAMHS).

Methods: This paper is based on a process evaluation examining how healthcare professionals experience both services, specifically focusing on their perceptions of young people's service engagement and recovery journey. Thirty-five semi-structured interviews were conducted with staff across various services, including 19 from ICCS and 16 other TAU services. A thematic decomposition analysis was conducted on the data, and specific themes relevant to staff experiences of young people's engagement with services and overall recovery were examined in-depth.

Results: Three main themes were observed in the HCP data (1. Relational Ecologies: barriers and enablers to engagement, 2. flexibility of approach amidst systemic pressures and 3. the web of trust in the relationship-building process). HCPs highlighted the necessity of developing trust and rapport through non-clinical engagement strategies, such as informal visits and personalised interactions. HCPs emphasised that without trust, treatment effectiveness diminishes, necessitating a tailored approach rather than a one-size-fits-all model. The flexibility in duration of treatment and methods of engagement were noted as crucial in accommodating individual client needs and fostering an open, trusting environment necessary for long-term recovery.

Conclusion: The findings highlight the vital importance of relational care models, especially ICCS, in addressing the complex needs of CYP. Flexible, family-centred approaches improve trust, engagement, and long-term recovery outcomes. Recommendations include tackling systemic barriers and expanding relational care models within CAMHS to meet increasing

mental health demands effectively. Further research should investigate scalable strategies for integrating these insights into wider mental health service frameworks.

Background

The prevalence of mental health challenges among children and young people (CYP) in high-income countries has increased steadily since 1995. Although Child and Adolescent Mental Health Services (CAMHS) have expanded, demand continues to exceed capacity [1]. Inpatient care has traditionally served as the main pathway for severe mental health conditions. However, recent evidence highlights the effectiveness of community-based alternatives in managing clinical risk and need outside institutional settings. Intensive Community Care Services (ICCS) have emerged as an alternative to hospital admission for CYP at high risk [2]. Expert consensus indicates that these approaches improve functional outcomes, reduce admissions, and preserve continuity in home and educational contexts [3].

Evidence from adult and youth services supports the value of intensive community models. Assertive Community Treatment (ACT) reduces hospital use and improves stability, symptom management, and life satisfaction [4,5]. Housing First, often combined with ACT, improves housing retention, quality of life, and recovery [6]. Functional ACT (FACT) enhances patient empowerment [7], while Interpersonal Community Psychiatric Treatment (ICPT) demonstrates effectiveness and cost-efficiency for long-term non-psychotic disorders [8]. Reviews show that home treatment, multisystemic therapy, and supported discharge achieve outcomes comparable or superior to inpatient care, while reducing costs and hospital stays and improving satisfaction [9]. These findings support a shift towards rights-based, recovery-oriented, community-centred care aligned with service-user preferences [10–12].

Research consistently shows that many CYP experience inpatient settings as restrictive and distressing [13,14]. Involuntary admissions associate with poorer experiences, higher suicide risk, dissatisfaction, increased readmission, and inequitable treatment of marginalised groups [13]. Post-discharge improvements tend to be modest, and staff evaluations of inpatient environments are often unfavourable [15]. These limitations have accelerated interest in community alternatives.

Emerging evidence indicates that community services can support diverse needs effectively. Forward Thinking Birmingham, developed from Youthspace, integrated assessment and treatment for young people aged 0–25. The model improved access, distributed clinical responsibility, and used digital platforms to enhance engagement. Although crisis and home treatment teams increased service reach, outcome improvements could not be confirmed due to data limitations [16]. This example illustrates both the potential and evaluation challenges of large-scale community initiatives.

Community-based organisations (CBOs) play a critical role in supporting marginalised populations. They provide safe spaces for identity expression, empowerment, and collective action, contributing to improved mental health among low-income, immigrant, and racially minoritised groups [17]. Community approaches for CYP promote personalised care, enhance engagement, and situate support within familiar environments, reducing stigma and encouraging help-seeking [18,19]. Their preventative focus enables early intervention and resilience-building before crises escalate [19].

Young people consistently identify continuity of care as central to therapeutic progress. Community settings often preserve continuity through more stable provider–service-user relationships [21,22]. Integration with local resources strengthens holistic support, and lower readmission rates further demonstrate effectiveness [13]. Cultural competence and family involvement improve relevance and responsiveness [14]. Addressing structural barriers and wider determinants of mental health, including poverty, housing, and social support, remains essential for sustainable prevention [23].

Despite this evidence, recent policy directions have deprioritised structural and community-level factors, even though they contribute substantially to psychological distress [20]. Community-based care re-centres these influences and facilitates contextually informed intervention [14]. Group therapy has also gained prominence, with evidence of reduced self-harm and suicidal ideation and lower crisis service use among adolescents [24]. Although NHS implementation plans prioritise community care for adults with severe mental illness [25], evidence on translating these models to CYP remains limited.

ICCS represents a targeted community-based alternative for CYP at risk of admission. Services typically operate in homes or schools, allowing young people to maintain daily routines while receiving intensive support [2]. Although the evidence base remains limited, existing studies suggest ICCS offers a viable alternative to treatment as usual, including during acute crises [15,26].

Despite growing evidence on effectiveness, limited attention has focused on staff experiences of delivering community-based care. Understanding professional perspectives is essential for identifying practical, relational, and organisational barriers and enablers. This study forms part of a wider RCT comparing ICCS with treatment as usual. Drawing on interviews with staff across both models, the analysis examines conditions that support or constrain effective delivery. Thematic analysis identifies patterns in professional engagement and perceived capacity to meet the needs of CYP and families.

Method

The qualitative data was part of a NIHR-funded RCT across ten NHS trusts in England and Wales, collecting data on CYP at risk and eligible for Tier 4 inpatient care. The trial compared ICCS with TAU to assess functioning, self-harm, education, and quality of life. Interviews evaluated perceived effectiveness, emotions, and impact on mental health for staff and service users. Thirty-five semi-structured interviews were conducted with HCP across various services, including intensive community care services (ICCS) (n=22) and other TAU services (Inpatient (n=4) & core CAMHS (n=9)). The HCPs range from consultant psychiatrists to nurse managers, registered nurses, clinical psychologists, and clinical care support workers. The recruitment of HCPs to be interviewed was conducted from 04/12/2022 to 30/01/2024.

Each interview lasted between 45 and 60 minutes. Before access to the HCPs was permitted, permission was granted by all local NHS, King's College London and London South Bank University research ethics committees. Written and verbal informed consent was given by all interviewed participants. Interviews were semi-structured and constructed with a specific set of themes derived from gaps in the literature around the contextual experiences of HCP delivering services in CAMHS, more significantly the ICCS intervention. However, sufficient flexibility in the interview structure was ensured to prompt participants to elaborate further on their

experiences, representing a greater depth of response. The interviews were transcribed verbatim, and the interview guide was developed based on input from a PPI group comprising young people who had previously used inpatient and core CAMHS community services.

Analysis

The validity of the findings was addressed using conventional qualitative procedures, including group analysis by key researchers and peer review, to ensure the analysis was sufficiently grounded in the data [27].

All authors participated collaboratively in the analytical process. Based on the aforementioned questions, the interview material was notated and coded, and the data were restructured into themes following an extensive round of discussion by the research team. This reorganised data was then examined alongside relevant literature to provide a contextual framework for the analysis. A thematic decomposition [28] approach was used to analyse the data thematically [14]. Furthermore, the aim was to identify processes through which mental health service use was enacted, understood, and experienced. This thematic decomposition was achieved by following several stages of analysis commonly found across all qualitative analysis analyses [29] including familiarisation with the data via repeated readings of the transcripts, generating initial codes by paying close attention to meanings embedded in every line of talk, and matching the initial codes together to form candidate themes and sub-themes, with the research questions guiding theme development. Each of the authors participated in discussions around whether the generated theme titles and definitions adequately captured the essence of the data. There was cross-validation at all analytical stages, including initial data coding, the expansion of coding into themes and the discussion of themes using key data. The interpretative process further involved exploring the implicit meaning of the material by prioritising a deeper understanding of the participants' experiences rather than a more descriptive reading.

Findings

Before discussing the main themes of this paper, we provide an overview of the general perspectives of the HCPs' experience across several services to provide a broader background context to the analysis.

General overview of services from the perspective of HCPs.

HCPs presented a range of their experiences across the services, particularly the ICCS, core CAMHS (TAU), and inpatient wards, reflecting both positive and negative aspects of care. ICCS is designed to provide flexible, community-based mental health support in environments such as schools or homes, enabling young people to maintain their daily routines while receiving care. This approach prioritises trust-building through informal engagement, like casual conversations or games, and relies on a single clinician for continuity, fostering stronger relationships with CYPs and their families. Family involvement is central to ICCS, with psychoeducation and empowerment initiatives helping parents regain autonomy while supporting their child's recovery. HCPs emphasized the importance of a tailored, culturally sensitive approach that adapts to the individual needs of service users, contrasting with the often-rigid frameworks of TAU. While TAU, which includes inpatient and Core CAHMS services, offers structured interventions, it faces challenges such as long waiting lists, reliance on medication before therapy, and limited opportunities for personalisation, often leaving CYP dissatisfied with their care [14].

Across both ICCS and TAU, healthcare professionals underscored the critical role of trust and engagement in achieving positive outcomes. Barriers such as systemic pressures, resource limitations, and lengthy wait times were identified as significant challenges, particularly in TAU services, which can hinder comprehensive therapeutic interventions. HCPs noted that ICCS's flexibility in scheduling, location, and approach often leads to better outcomes by addressing the unique needs of young people and their families. They also highlighted the importance of empowering parents and addressing family dynamics to ensure sustained support for CYP during recovery. However, systemic demands often compromise the quality of care in both models, leading to a reliance on quick fixes like medication instead of holistic and relational approaches. These insights reinforce the need for more adaptable, relationship-focused mental health services to support young people and their families effectively.

Main analysis

The analysis revealed three main themes related to the in-depth experiences of healthcare professionals delivering services in the ICCS and TAU settings. The themes include "Relational Ecologies: barriers and enablers to engagement", "Parental role in the ecosystem: enskilling parents", "The web of trust in the relationship-building process.   Participants were asked about their experience of supporting young people within their services, including some explicit references to the barriers and facilitators related to delivering their services to CYP. However, the data revealed how barriers and enablers were organised thematically around issues of relationality.

Relational Ecologies: barriers and enablers to engagement.

All HCPs characterised their experiences of working with CYPs as interactions that were part of a broader relational ecosystem. The term ecosystem here refers to the comprehensive network of therapeutic relationships and interactions present between stakeholders, including healthcare professionals, peers, and family members. Within the context of our study, there are numerous interconnected and interdependent entities present across the different services within CAMHS. According to the accounts provided by HCPs, these interdependencies among the various entities (HCPs, peers, family/guardian, and other agencies) have been identified as significant factors influencing young people's engagement with support. This engagement encompasses the initial help-seeking phase and extends to the CYPs ongoing involvement with the services offered. A key relational form influencing service engagement was reported to be the family network, specifically familial expectations and anxieties.

You often have to tolerate lots of that reaction, the disappointment, the distress and you quite often get told you are not doing a good job, not doing enough, being neglectful (HCP Inpatient).

For example, somebody that's being admitted to a ward, we're still involved and we'll meet with families, one on one, without the young person, we'll meet with the family and get their perspective and relay what we can do to navigate the issues that they've got so that they feel more comfortable (HCP ICCS).

The critical role of trust between HCPs and families in delivering effective mental health care was emphasised. HCPs highlight reflective practice, showcasing their awareness of the need to ensure parents feel understood and confident in the care provided for their child. This self-awareness indicates that trust is not assumed but actively cultivated through thoughtful communication and consistent engagement. Without such trust, families are less likely to feel reassured or collaborate effectively with providers.

Additionally, as described above, HCPs frequently encounter disappointment and distress from families. The emotional toll of addressing these reactions underscores the intense relational labour required in mental health care. Parents' dissatisfaction often stems from unmet expectations or long-standing frustrations with the healthcare system.

There is a ripple effect on how these feelings of disappointment, fuelled by prolonged waiting times, can extend to the CYP, potentially impacting their engagement with services. This dynamic reveals the cascading effect of systemic inefficiencies on relational trust and therapeutic outcomes.

Parents are often coming from that place of disappointment with the service considering the length of time they have spent on the waiting list before getting some treatment. The disappointment often transmits to their wards and how ready they are to engage with the service (HCP Inpatient).

Past negative experiences with mental health services are identified as significant barriers to family engagement, as noted in the quote by (HCP Inpatient) above. Even when HCPs offer a more collaborative, tailored approach, families may remain sceptical, expecting more of the same inadequate care. This scepticism can hinder the establishment of trust and delay meaningful engagement, requiring HCPs to invest additional time and effort in rebuilding relationships.

Whilst YPs are then engaged with the services, there are descriptions of the expectations of the relationships (formal and informal) that they have with the HCPs within the services. HCPs emphasise the role of relationships and trust in effective service delivery.

We take our time to explain to the young people that we cannot force them to do anything and that our purpose is to try and put them in a better place mentally from where they are, we say to them the reason why we can't force you to do anything is because they're difficult to engage and if they feel they're being pressured in any shape or form they're just going to back off even more (HCP ICCS).

The importance of a non-coercive approach when working with young people who are often difficult to engage was discussed, explaining to CYP that they are not being forced into treatment, as any perceived pressure could lead to disengagement. This reflects an understanding of the autonomy and agency of young people, which is critical for fostering trust. By adopting a patient-centred strategy, the HCP prioritises creating a safe, judgment-free environment where YP feel empowered rather than controlled. This aligns with trauma-informed care principles, which advocate minimising power imbalances and respecting the client's pace [30].

Additionally, HCPs highlight the quality of relationships between young people, their families, and service providers is directly tied to positive outcomes. By emphasising "really strong relationships," the HCP connects engagement and trust-building with better mental health progress. This insight reflects the relational nature of ICCS, where the human connection between the client and the care team is foundational for effective intervention.

If I'm honest with you, that's where the really good work is done, and we tend to find that outcomes improve when we have really strong relationships between the young person, the family, and those involved in their care (HCP ICCS).

And with ICCS, because it's just the one clinician that's the main contact you can develop a better relationship and hopefully have more influence (HCP ICCS).

I think relationships are important because especially if you're in a crisis situation, you want to be working with someone that you feel understands you, that you don't have to give your whole life story to again and, also they can see the progression (HCP ICCS).

In addition, there were discussions around the role of peer relationships developed during their engagement with services. HCPs recognise that while peers can serve as a source of emotional support, they may also inadvertently perpetuate harmful behaviours, such as self-harm, through social learning or normalisation [31]. This dynamic reflects the vulnerability of adolescents who often turn to peers for help but lack the guidance of healthcare professionals equipped to address these challenges effectively.

"So, some young people only speak with peers and they end up learning behaviours that cause harm as well, sometimes we have groups of ..., ... in person and you learn that they have a group of friends and a few of their friends' self-harm, so they learn the way that they find helpful to cope with the symptoms (HCP ICCS)".

"Sometimes the tricky thing is that some young people confide in their peers and that's not always helpful because to tell another young person a secret or get support from another young peer who is also maybe struggling in adolescence is very difficult because obviously, they're not able to support them as much as what they might need (HCP Core CAMHS)".

According to the data presented above, there appears to be a risk of maladaptive coping mechanisms spreading within peer groups, particularly among CYP who may share similar struggles. Discussions reveal how peer dynamics can influence behaviour in ways that hinder recovery, as adolescents may adopt unhealthy strategies observed in others. Moreover, the limitations of peer support were highlighted, pointing out that young people who are themselves struggling may lack the emotional resources or maturity to provide adequate help. This can lead to a cycle of unmet needs and reinforce feelings of isolation or distress. This suggests the importance of fostering safe, supportive environments where professional guidance complements peer interactions, helping young people develop healthier coping mechanisms and break cycles of harm within their social networks.

Parental role in the ecosystem: enskilling parents

This section focuses more on the role of parents and families and their contribution to facilitating engagement with services. This includes parents acting as an avenue for help-seeking for mental health concerns or determining how well young people engage with services. More specifically, HCPs acknowledged the need to upskill parents to support the CYP in their care more efficiently. Harden [33] highlighted that HCPs often undermine parents' confidence as parents are often accused by HCPs of overacting or being overly dramatic or anxious about their child's health concerns. Instead of recognising and validating parents' worries, HCPs may label concerns in ways that lead to parents feeling belittled or dismissed, potentially undermining their ability to trust their instincts and seek further medical advice when necessary. As a result, the relationship between parents and healthcare providers may become strained, which can ultimately affect the quality of care and support that the child receives.

"You have to try to always think of the young person's best interests and balancing how the parents come into that, and I agree, sometimes it might be that they are not on the same page, so it's trying to learn how they can be in the same place, but not in a family therapy kind of way because we don't do that in an outreach thing, just trying to see how the parents can understand their needs a bit more, and trying to see how both sides can communicate better (HCP ICCS)".

While the CYP's interests are considered a priority, the entire relational ecosystem, requires careful management in order for the intervention to succeed. This includes managing the knowledge and skills parents bring to the service and ensuring these are developed and parents empowered:

"So, it's skilling up the parents as well and developing an understanding and formalising the difficulties (HCP ICCS)".

"Parents will be given psychoeducation about mental health illness so that they feel more comfortable speaking to the young people about that. (HCP Urgent care team)"

Upskilling parents with the skills that empower them to take an active role in the management of their child's recovery process seems vital.

“We sometimes find that families will say my young person wants to be going out, can I let them, and we say you are the parent, so give them back the power, because when someone is in crisis we, as professionals, do go in and we do take a lot of the power away from parents and we can become quite restrictive initially for safety reasons, but then we have to bear in mind that it’s the family that’s living with this young person and we have to empower them to look after that young person. (HCP ICCS)”

“I think with education we need to help families recognise that actually mental health struggles are multi-determined and they don’t just come from one place, and I suppose one of my fears is that with labels, once the labels start to get attached, some families, not because they’re trying to blame one thing, but I think it’s because they just don’t understand all the different factors that have an influence on young people’s or on anybody’s mental health, so I think we need a more open education type system, a non-blaming education system around mental health and how everybody can influence positive mental health by the way we interact. (HCP ICCS)”

HCPs highlighted the importance of empowering parents to take charge of their children’s well-being, particularly during times of crisis. They acknowledged professionals' role in taking control for safety reasons but highlighted the need to empower families to ultimately care for their young person in the longer term, involving them right from the start. They also touched on the complexity of mental health struggles and the potential drawbacks of attaching labels to them. This suggests the need for a more comprehensive and non-blaming education system that alerts families to the various factors influencing mental health and how everyone can contribute to positive mental well-being through positive relationships. Together, the ICCS philosophy of relational care is highlighted here, which seems to prioritise families as central actors in a young person’s mental health journey. Empowering families requires healthcare professionals to step back and equip parents with knowledge and confidence. This approach also reflects an acknowledgement of systemic power dynamics in healthcare, where professionals must navigate between being agents of immediate action and facilitators of long-term family-led care.

To the role of parents in the care and support of young people (YP) with mental health concerns is considered by HCPs to be crucial. Beyond educating and empowering parents/guardians, HCPs emphasise the role of developing a relationship with family, especially in ICCS, to improve engagement levels.

I think it's always been relatively straightforward, we will be working with children that don't want us there, but we will be working with parents that do, and so our ability to get them on board is relatively straightforward (HCP ICCS).

even the parents have to do a lot of parent work where the parent would call me every day and say we'll have a chat about what's going on, and I think they found that a bit comforting, and obviously they needed that, but I guess it's not attainable for a long period of time, so we had to work on independence quite a few times (HCP ICCS).

Often having conversations with the parents, knowing that they're in a position where they probably do feel like they're failing and that they are responsible (HCP ICCS).

HCPs recognise the initial control taken by professionals for safety reasons but stress the importance of ultimately empowering families to care for their YP. Furthermore, there is a call to integrate the family's role as a perduring support system with the aim of creating a collaborative framework that promotes resilience for both the YP and their caregiver.

The web of trust in the relationship-building process.

HCPs play a crucial role in the relational ecosystem, which is the central theme discussed here. The length of time required to build meaningful therapeutic relationships and their intersection with gaining the trust of parents and young people were mainly discussed by the HCPs. Whilst HCPs appeared to be time pressured to engage with YP in the various services, gaining trust seems vital to the dynamic relationship between HCPs and YP and well worth the temporal investment [33].

"Lots of our service delivery is about the relationship and how sometimes you need to spend quite a long time than you do in other services building up the relationship, and sometimes

it's ..., sometimes it's coffee or bubble tea, or sometimes playing a game with them during lunchtime when they are in the day service and that's part of the relationship building up. (HCP ICCS)"

"I think in our service, we are actually meant to be a short-term service of 12 weeks and often we will extend beyond that because of the lack of engagement and so in terms of 12 weeks I would say that isn't enough but we often and do extend beyond that. I have worked with someone for nearly a year before so there is flexibility, we have that luxury of flexibility in that we don't have a waiting list so we can do that. (HCP Inpatient)"

"The last thing they want to do is go into a clinic and say here's all my stuff. And to do that takes time, it takes the person's trust, it takes the ability for them to realise that I'm not going to get in trouble for this stuff and actually, I might get some help for it, and then tailoring that help as much as you can for whatever is going on for them. (HCP Core CAMHS)"

"Then I think in our work it's really not having an agenda, so at the moment we're very lucky, we don't have targets we have to meet, there's no KPIs, there's none of this that we are stuck to in terms of stuff we have to get done, we are getting them done, but we're getting them done in our own way which I think is another really helpful thing (HCP ICCS)".

Based on the above extracts, HCPs emphasise the significance of building strong relationships in service delivery. They highlight the importance of spending time and engaging in various informal relational activities, such as having coffee or playing games, to develop a strong rapport with clients, prioritising personalised and relational aspects of service delivery.
In addition, the flexibility of service duration was also described as crucial, extending beyond the standard 12 weeks to accommodate the need for continued engagement and support. This call for flexibility recognises the inadequacy of a fixed time frame in addressing clients' needs. Time, trust, and tailored support are required for clients to open up and seek help. The need for flexibility in approach underscores the need for a non-clinical, more informal, trusting

environment and the importance of personalised assistance to address clients' unique circumstances.

"There are so many people, so many different parts. Somewhere between three and five percent of the people come from CAMHS, I think that's the amount that we get per year, so we are only dealing with a very, very small percentage of people so that means that so many children who go into CAHMS have their therapies and come out fine; that's a lot. When I'm working with someone, I am working with horrendous stuff so home treatment is when working in a clinic hasn't worked, talking therapies haven't worked, DBT or whatever therapies haven't worked and when we don't see things working in an environment like a clinic perhaps we can give them home treatment and come out and work with you there. So, we hit that 3% of people with a comfortable place to work, a more personable 'let's have a look at this, let's have a look at that' mentality and then be able to draw in the therapies, sometimes without the kids even realising that they're doing that (HCP ICCS)".

"The young person and the families, in general, they're not going to respond to any treatment if they don't engage, they don't trust. So that is pretty key: it's very important for them to trust us. (HCP ICCS)"

"I think the relationships, they have to trust you and you have to trust them as well and that's the basic relationship that you have to have with them and then develop the engagement. It may be that I go to them, I go to their house the first day and I don't do a medical assessment or anything, I just go there to engage with them and I may have to go a few times to be able to engage with them because they may not trust you – who is this person coming to my house who I've never met? So, that is why it is really important that we try to develop that engagement as soon as possible to be able to provide the service. (HCP ICCS)"

These quotes emphasise the importance of building trust and establishing a strong relationship between HCPs and CYPs and their families. Particularly in the community setting (ICCS), HCP stressed that without trust and engagement, any treatment or service provided would not be

effective. They highlight the need to invest time in developing trust, even if it means making multiple visits without immediately focusing on any formal medical assessments. Building trust and engagement was seen as the foundation for successful healthcare outcomes
and the flexibility built into the approach was seen as a key element in strengthening the cord of trust:

"Some people work more flexibly; some people work more proactively, and other people might not. It's a clinical style: different people have a different approach (HCP Core CAMHS)".

"That they feel listened to and understood, they don't feel you are trying just to go through a checklist of items because they are all so used to that because usually the young people that come to us are not new in CAMHS (HCP ICCS)".

"Some might like a certain approach; others might not like that: so, a professional being able to adapt to that (HCP ICCS)".

"I never feel old, so when I've got someone in the car or if I'm in someone's house or if I've taken them to a café I chat about what they want to chat about to start off with, I ask them from their point of view and if I don't understand something or don't know something I will say I don't understand that, what is the attraction to that, why do people like that band. And if they're talking about something like Love Island I can turn round and say from my point of view materialism, oh that's pretty bad, but you lot seem to like it, why is that, and they will be able to give you the answers, and so you can work out where their moral values are, you can work out almost what lessons they've missed (HCP ICCS)."

"I once built a rapport with a boy who had autism and refused to speak to every other professional from our team when he was assessed previously, and I used 15 minutes of my appointment speaking about a Michelin star restaurant he went to in London and about food and how different flavours can make a meal better etc, so that is how I developed a rapport (HCP CAHMS Rapid response team)".

The data extracts offer insights into the diverse and personalised approaches employed by HCPs. They underscore the significance of flexibility, active listening, adaptability, and rapport-building in clinical practice. The emphasis on recognising individual differences, engaging in empathetic dialogue, and tailoring interactions to meet the unique needs of individuals highlights the personalised and human-centric nature of mental health care.

In inpatient settings, they have got loads of different people to talk to and people offer different things to them and they are generally quite good at going to different people for different things, based on what they get from them but I think sometimes it is a bit confusing to them if they feel they have heard different things from different people, there is the potential for splitting or whatever. I think that even though I feel like everyone can have an input in decision making, I think often the young people feel that everything goes to the consultant and she's the only person who has the power really to do anything (HCP inpatient).

The above extract signals an acknowledgement of the variety of professionals available to service users, which can be beneficial as individuals seek different types of support from various team members particularly in inpatient settings. This diversity is important because it allows patients to address specific needs by reaching out to those best suited to assist them. However, this also points to a significant issue regarding the potential for confusion when patients receive differing information from multiple sources. This inconsistency can lead to feelings of fragmentation or "splitting," where patients may struggle to reconcile conflicting messages from different HCPs.

Additionally, other members of the clinical team could take a more proactive role in decision-making rather than deferring to the lead consultant. Empowering all members of the team to make decisions, like in the ICCS model, could enhance collaboration and patient autonomy, allowing for a more inclusive approach to care. This collaborative decision-making model can diminish confusion for the patients by providing them with consistent messaging and support from a cohesive team, improving their overall experience and satisfaction with the care process.

Discussion

This study explored the perspectives of HCP delivering intensive community care services (ICCS) versus TAU in CAMHS across multiple UK sites. The findings reveal a nuanced balance of barriers and enablers that influence effective service delivery. ICCS staff emphasised key enablers such as the flexibility to deliver care in homes and schools, strong family engagement, and a team-based approach that allowed holistic, context-driven interventions. Staff described how providing treatment in a young person's everyday environment helped maintain normal routines and family connections, aligning with known advantages of community-centred care (e.g. preventing isolation from school/family and enabling holistic assessment) [2]. They felt this approach fostered trust and sustained improvements, echoing published reports that ICCS settings can increase the likelihood of sustainable change in outcomes. Notably, HCPs gave examples of working creatively with families, for instance, scheduling sessions around school or meeting in informal settings, which they saw as critical facilitators of engagement. These accounts underscore how ICCS leverages the home and community context as a therapeutic asset, consistent with literature highlighting that community-based treatment can reintegrate young people early and avoid the disruptions of inpatient admission [2]. In turn, TAU staff often working in traditional inpatient or clinic settings described different challenges. They noted barriers like the fragmentation of care when transitioning patients back home and the limit of short inpatient stays in addressing complex family dynamics. Some TAU practitioners expressed frustration that, without an intensive community option, young people "bounced" between hospital and home with insufficient support. This perspective reinforces why ICCS models were developed to fill the gaps left by standard services [2]. Indeed, our findings illustrate that many barriers in TAU (e.g. abrupt discharge, loss of continuity) are the very issues ICCS is designed to mitigate by providing extended, home-based support.

Despite the strengths of ICCS, HCPS in both TAU and ICCS candidly discussed significant barriers to effective delivery. Resource and capacity constraints were a prominent theme. ICCS teams often carried small caseloads by design, yet staff still reported high workload intensity given the complexity of cases and the 24/7 nature of support. Some ICCS interviewees described the emotional toll of managing risk in the community; responding to crises at odd hours was seen

as necessary but taxing, raising concerns about burnout. Likewise, TAU staff highlighted chronic understaffing and bed shortages that hampered their ability to provide high-quality care – an issue consistent with wider systemic problems noted in prior analyses (e.g. stakeholder reports of an overstretched CAMHS system). Another barrier was inter-agency collaboration. ICCS staff sometimes struggled to coordinate with schools, social services, or inpatient units, especially when roles were unclear or when colleagues in traditional settings were sceptical of the community approach. This finding echo previous observations that ICCS and similar teams often operate in isolation without shared protocols, underscoring calls to better integrate these services into the broader system [36]. Safety and risk management were identified as a further challenge. TAU staff were concerned about certain high-risk CYPs being managed at home, while ICCS staff acknowledged feeling “on their own” when handling severe crises in the field. Importantly, HCPs described how strong team support and supervision were essential enablers to overcome these stresses. For example, regular team debriefs after crises and a culture of collective problem-solving helped staff feel more confident and supported. This points to the critical role of team cohesion and training as enabling factors in both models. These qualitative insights build on and enrich the existing research on intensive community care. Previous studies have mainly quantified outcomes, showing that intensive community treatments can reduce hospital use and improve patient satisfaction relative to usual care [15]. Our findings help explain how those outcomes may be achieved. For instance, staff attributed reduced admissions to proactive work in the community, “keeping the young person safely at home,” which aligns with evidence that ICCS is associated with fewer and shorter hospital stays. They also observed better engagement and trust from families, echoing reports of greater patient/family satisfaction under community-based models. By detailing the mechanisms (e.g., building rapport in familiar environments, addressing problems in real-time at home), this study provides context for the positive quantitative outcomes noted elsewhere [15,26]. Our findings support the view that intensive community care can be effective and person-centred, but its success depends on implementation factors. When those factors are favourable (adequate staffing, inter-agency support, skilled clinicians), ICCS fulfils its promise of comprehensive,

home-based care. When they are not, even a well-intended ICCS can fall short of a reality that highlights why ongoing evaluation and refinement of these services are critical.

Strengths and Limitations

This study has several strengths. By capturing first-hand accounts from staff in three distinct service models, it provides a rich, comparative perspective that is rarely seen in the literature. The use of thematic decomposition enabled a thorough analysis of the meanings associated with relational practices. The in-depth thematic analysis yielded insights into the day-to-day facilitators and obstacles in service delivery, offering depth and context to complement the Trial element. Another strength was the inclusion of participants from multiple professional backgrounds (e.g. nurses, social workers, psychiatrists) across ICCS and TAU. This diversity increased the breadth of perspectives, enhancing the credibility of the themes by showing they were not isolated to one role or site. We also explicitly examined two contrasting models (ICCS vs. TAU), which strengthens the analysis by allowing us to distinguish which challenges are model-specific and which are more general in children's mental health services.

However, the study only captured staff perspectives. It did not include direct accounts from young people or families, which limited the ability to triangulate the findings fully. However, clear points of comparison can be made with a separate paper reporting data with young people from this study [37].

Furthermore, interviews were conducted within the framework of an RCT, which may not entirely reflect real-world service delivery outside of research settings.

Implications for Practice

These findings suggest that relational models such as ICCS should be further integrated into mainstream CAMHS delivery. Specifically, the development of ICCS should focus on building an effective support system for young people. It is essential to prioritise flexible, individualised approaches that accommodate their preferences for time, location, and method of engagement. Strengthening staff training in trauma-informed, non-coercive, and culturally sensitive care practices is crucial for providing the best possible support. Additionally, actively

involving parents and carers through psychoeducation will empower them as partners in the recovery process, further enhancing the effectiveness of interventions.

To simplify access to care, developing strategies that address systemic barriers is vital, particularly by reducing waiting times for therapy and equipping young people with clearer and more transparent navigation tools for services. Managing peer influences is also important; implementing professionally supported peer programs can help prevent the spread of maladaptive behaviours among youth.

Moreover, it is crucial to protect staff from relational fatigue by ensuring manageable caseloads and providing reflective supervision spaces that focus on the relational aspects of their work. Building these relational infrastructures necessitates a concurrent commitment to addressing broader systemic constraints, such as workforce shortages and rigid care pathways, to ensure long-term sustainability and effectiveness of the support provided.

Conclusion

This study provides compelling evidence that relational care, underpinned by trust, flexibility, and family engagement, is central to successful community-based mental health interventions for CYP. ICCS offers a promising model by embedding these relational principles into service delivery. However, systemic challenges, including workforce limitations and service pressures, continue to undermine these efforts. Future service models must combine relational excellence with structural reform to ensure that all young people accessing mental health services experience care that is personalised, empowering, and effective. Further research exploring young people's direct experiences and the scalability of relational care models will be critical for informing national service redesign.

Declarations

Ethical Approval

Ethical approval was obtained from the West Midlands and Black Country Research Ethics Committee and the Health Research Authority in the UK, REC reference: 20/WM/0069. The trial was conducted in compliance with the principles of the Declaration of Helsinki (1996), the

principles of GCP and in accordance with all applicable regulatory requirements including but not limited to the UK policy framework for health and social care research. The Sponsor trust granted Local Research and Development approval. Informed consent was obtained from all participants.

Funding

This study was part of a randomised controlled trial, funded by the NIHR HTA Programme (Ref: NIHR127408). The funder plays no role in the trial design, delivery, and interpretation of data, the writing of the report; and the decision to submit the report for publication.

Trial registration

The IVY trial was registered on April 29, 2020, ISRCTN number: ISRCTN42999542.

Availability of data and materials

Anonymised data will be available on request.

Conflict of Interest

The authors declare that the research was conducted in the absence of any commercial or financial relationships that could be construed as a potential conflict of interest.

Authors' Contributions

OA, PR, and BHCW contributed to the main manuscript as well as data collection. All authors contributed to recruitment and critical review.

Consent for publication

Not applicable. All personal identifiable information has been excluded in the manuscript.

List of References

1. Pitchforth, J., Fahy, K., Ford, T., Wolpert, M., Viner, R. M., & Hargreaves, D. S. (2018). Mental health and well-being trends among children and young people in the UK, 1995–2014: analysis of repeated cross-sectional national health surveys. *Psychological Medicine*, *49*(08), 1275–1285. https://doi.org/10.1017/s0033291718001757
2. Keiller, E., Masood, S., Ben Hoi-Ching Wong, Avent, C., Bediako, K., Bird, R. G., Boege, I., Casanovas, M., Dobler, V., James, M. L., Kiernan, J. G., María Martínez-Hervés, Ngo, T., Pascual-Sánchez, A., Izabela Pilecka, Plener, P. L., Prillinger, K., Isabelle Sabbah Lim, Saour, T., & Singh, N. (2023). Intensive community care services for children and young people in psychiatric crisis: an expert opinion. *BMC Medicine*, *21*(1). https://doi.org/10.1186/s12916-023-02986-5
3. Hayes, D., et al. (2023). Intensive community care services for children and adolescents: Consensus statement. *BMC Medicine*, 21(1), 35.
4. Stein, L. I., & Test, M. A. (1997). The evolution of the assertive community treatment model. *Community Mental Health Journal*, 33(6), 527–534.
5. Bond, G. R., & Drake, R. E. (2015). The critical ingredients of assertive community treatment. *World Psychiatry*, 14(2), 240–242.
6. Stergiopoulos, V., et al. (2019). The effectiveness of Housing First in five Canadian cities. *BMC Public Health*, 19, 1471.

7. Mulfinger, N., et al. (2024). Functional ACT-based interventions and patient empowerment: A German multicenter RCT. *BMC Psychiatry*, 24(1), 78.

8. van Veen, M., et al. (2021). Interpersonal Community Psychiatric Treatment: A randomized controlled trial. *BMC Psychiatry*, 21, 150.

9. Cottrell, D. J., et al. (2020). A systematic review of intensive community treatment for children and young people. *Journal of Child Psychology and Psychiatry*, 61(1), 18–29.

10. Killaspy, H., et al. (2022). Supported accommodation for people with serious mental illness: Systematic review. *World Psychiatry*, 21(2), 233–254.

11. NHS England. (2024). Guidance to integrated care boards on intensive and assertive community mental health care.

12. World Health Organization. (2023). Guidance on community mental health services: Promoting person-centred and rights-based approaches.

13. Bartl, G., Stuart, R., Ahmed, N., Saunders, K., Loizou, S., Brady, G., Gray, H., Grundy, A., Jeynes, T., Nyikavaranda, P., Persaud, K., Raad, A., Foye, U., Simpson, A., Johnson, S., & Lloyd-Evans, B. (2024). A qualitative meta-synthesis of service users' and carers' experiences of assessment and involuntary hospital admissions under mental health legislations: a five-year update. *BMC Psychiatry*, *24*(476). https://doi.org/10.1186/s12888-024-05914-w

14. Reavey, P., Poole, J., Corrigall, R., Zundel, T., Byford, S., Sarhane, M., Taylor, E., Ivens, J., & Ougrin, D. (2017). The ward as an emotional ecology: Adolescent experiences of

managing mental health and distress in psychiatric inpatient settings. Health & Place, 46, 210–218. https://doi.org/10.1016/j.healthplace.2017.05.008

15. Ougrin, D., Corrigall, R., Stahl, D. *et al.* Supported discharge service versus inpatient care evaluation (SITE): a randomised controlled trial comparing effectiveness of an intensive community care service versus inpatient treatment as usual for adolescents with severe psychiatric disorders: self-harm, functional impairment, and educational and clinical outcomes. *Eur Child Adolesc Psychiatry* **30**, 1427–1436 (2021). https://doi.org/10.1007/s00787-020-01617-1
16. Birchwood, M. J., Street, C., Singh, S. P., Lamb, C., Anderson, Y., Warner-Gale, F., Sedgewick, J., Thompson, A. D., & Upthegrove, R. (2018). *Impact and process evaluation of Forward Thinking Birmingham, the 0-25 Mental Health Service : Final Report.* .University of Warwick ; University of Birmingham ; GIFT (Great Involvement Future Thinking) ; CLAHRC-WM. https://wrap.warwick.ac.uk/id/eprint/100545/
17. Bloemraad, I., & Terriquez, V. (2016). Cultures of engagement: The organizational foundations of advancing health in immigrant and low-income communities of color. *Social Science & Medicine*, *165*, 214–222. https://doi.org/10.1016/j.socscimed.2016.02.003
18. McGorry, P., Bates, T., & Birchwood, M. (2013). Designing youth mental health services for the 21st century: examples from Australia, Ireland, and the UK. *British Journal of Psychiatry*, *202*(s54), s30–s35.

19. Vyas, N. S., Birchwood, M., & Singh, S. P. (2014). Youth services: meeting the mental health needs of adolescents. *Irish Journal of Psychological Medicine*, *32*(1), 13–19. https://doi.org/10.1017/ipm.2014.73

20. Callaghan, J. E., Fellin, L. C., & Warner-Gale, F. (2016). A critical analysis of Child and Adolescent Mental Health Services policy in England. *Clinical Child Psychology and Psychiatry*, *22*(1), 109–127. https://doi.org/10.1177/1359104516640318

21. Wong, B. H. C., Chu, P., Calaminus, P., Lavelle, C., Refaat, R., & Ougrin, D. (2024). Association between continuity of care and attendance of post-discharge follow-up after psychiatric emergency presentation. npj mental health research, 3(1), 5.

22. Naert, J., Roose, R., Rapp, R. C., & Vanderplasschen, W. (2017). Continuity of care in youth services: A systematic review. *Children and Youth Services Review*, *75*, 116–126. https://10.1016/j.childyouth.2017.02.027

23. Clement S, Schauman O, Graham T, et al. What is the impact of mental health-related stigma on help-seeking? A systematic review of quantitative and qualitative studies. *Psychological Medicine*. 2015;45(1):11-27. doi:10.1017/S0033291714000129

24. Gerstl, B., Opoku Ahinkorah, B., Nguyen, T.P., Rufus John, J., Hawker, P., Winata, T., Brice, F., Bowden, M., & Eapen, V. (2024). Evidence-based long-term interventions targeting acute mental health presentations for children and adolescents: systematic review. Frontiers in *Psychiatry, 15*(1). https://doi.org/10.3389/fpsyt.2024.1324220

25. NHS England. (2022). *NHS mental health implementation plan 2019/20–2023/24*. NHS England. https://www.england.nhs.uk/wp-content/uploads/2022/07/nhs-mental-health-implementation-plan-2019-20-2023-24.pdf

26. Ougrin, D., Corrigall, R., Poole, J., Zundel, T., Sarhane, M., Slater, V., Stahl, D., Reavey, P., Byford, S., Heslin, M., Ivens, J., Crommelin, M., Abdulla, Z., Hayes, D., Middleton, K., Nnadi, B., & Taylor, E. (2018). Comparison of effectiveness and cost-effectiveness of an intensive community supported discharge service versus treatment as usual for adolescents with psychiatric emergencies: a randomised controlled trial. *The Lancet Psychiatry, 5*(6), 477–485. https://10.1016/S2215-0366(18)30129-9
27. Creswell, J., & Miller, D. J. (2000). Determining validity in qualitative inquiry. Theory Into Practice, 39, 123–130. https://doi.org/10.1207/s15430421tip3903_2
28. Stenner, P. (1993). Discoursing jealousy. In E. Burman, & I. Parker (Eds.), Discourse analytic research (pp. 114–134). Routledge.
29. Willig, C. (2008). An introduction to qualitative research in psychology: Adventures in theory and method. London: Sage.
30. Miller, N., Nair, S., & Majumder, P. (2023). Is it 'just' trauma? Use of trauma-informed approaches and multi-agency consultation in mental healthcare of looked after children. *BJPsych Bulletin, 47*(6), 337. https://10.1192/bjb.2023.3
31. Schwartz-Mette, R. A., Shankman, J., Dueweke, A. R., Borowski, S., Rose, A. J., & Schwartz-Mette, A. (2020). Supplemental Material for Relations of Friendship Experiences With Depressive Symptoms and Loneliness in Childhood and Adolescence: A Meta-Analytic Review. *Psychological Bulletin,* https://10.1037/bul0000239.supp
32. Harden J. "Uncharted Waters": The Experience of Parents of Young People With Mental Health Problems. *Qualitative Health Research*. 2005;15(2):207-223. doi:10.1177/1049732304269677

33. Hardin, H. K., Bender, A. E., Hermann, C. P., & Speck, B. J. (2021). An integrative review of adolescent trust in the healthcare provider relationship. *Journal of Advanced Nursing, 77*(4), 1645–1655. https://10.1111/jan.14674

34. World Health Organisation. (2022). *MENTAL HEALTH AND COVID-19: EARLY EVIDENCE OF THE PANDEMIC'S IMPACT: SCIENTIFIC BRIEF, 2 MARCH 2022.* .States News Service.

35. Adu, M. K., da Luz Dias, R., Obuobi-Donkor, G., Ezeanozie, N., Sridharan, S., Morrison, J., Simon, P., Taylor, B., MacKinnon, M., Gossen, S., Awara, M., White, M., Shalaby, R., Agyapong, B., Eboreime, E., Wang, J., Feng, C., Wozney, L., Koto, P., . . . Agyapong, V. I. O. (2024). Reducing wait times and avoiding unnecessary use of high-cost mental health services through a Rapid Access and Stabilization Program: protocol for a program evaluation study. *BMC Health Services Research, 24*(1), 247. https://10.1186/s12913-024-10697-7

36. Cooper, M., Evans, Y., & Pybis, J. (2016). Interagency collaboration in children and young people's mental health: a systematic review of outcomes, facilitating factors and inhibiting factors. *Child: care, health and development*, *42*(3), 325–342. https://doi.org/10.1111/cch.12322

37. Paula Reavey, Opeyemi Atanda, Ben Hoi-ching Wong et al. Relational dynamics and experiences of inpatient and intensive community care for children and young people: matters for service engagement and recovery, 25 August 2025, PREPRINT (Version 1) available at Research Square [https://doi.org/10.21203/rs.3.rs-5574483/v1]
