# Supplementary material for: Relational care in community mental health: Evaluating staff experiences in Intensive Community Care Services (ICCS) vs treatment as usual
Source: PLoS One. 2026 Sep 9;21(9):e0356608. doi: 10.1371/journal.pone.0356608 (PMC13557356; doi:10.1371/journal.pone.0356608)
Supplement: Supplementary file 3 [file pone.0356608.s004.docx]

Dear Editor,

We would like to thank you and the reviewers for the time and effort devoted to evaluating our manuscript. We are grateful for the constructive and thoughtful comments, which have been invaluable in strengthening the manuscript's clarity, methodological transparency, and overall contribution.

In response to the reviewers' comments and suggestions, we have undertaken a thorough revision of the manuscript. A detailed point-by-point response is provided below.

| **Reviewers’ comments** | **Responses** |
| --- | --- |
| **Reviewer 1** | |
| The aim needs to be clearly stated as the statement of aim is overshadowed by a RCT which was a predecessor | Thank you for this helpful suggestion. We have revised the Introduction to distinguish more clearly between the wider randomised controlled trial and the present qualitative study. Specifically, we have added a dedicated study aim at the end of the Introduction, clarified that this paper reports findings from the trial's qualitative component, and revised the opening of the Methods section to reinforce this distinction. These changes ensure that the focus on healthcare professionals' experiences of delivering ICCS and TAU is clear from the outset. |
| In the methodology- sampling technique and how sample size was finalized is lacking. | Thank you for highlighting the need for greater methodological detail. We have expanded the Participants section to clarify that a purposive sampling strategy was used to recruit healthcare professionals with direct experience of delivering ICCS or TAU across participating CAMHS services. We have also described the recruitment procedures and provided a rationale for the final sample size. Consistent with qualitative research principles, the sample size was guided by information power (Malterud et al., 2016), reflecting the focused research aim, the specificity of the participant group and the richness of the data collected, rather than statistical representativeness. |
| The interview guide can be included | The full interview guide is now available as Supplementary File 1. |
| In the analysis - the method of analysis whether manual or software is not mentioned | We have added a participant characteristics table including professional role, service type, years of experience, and geographical location to improve transparency and transferability. |
| How codes were generated can be described | We have expanded the analytic procedures section to provide a clearer account of how initial codes were generated, refined, and developed into themes. |
| The findings' part includes themes but not subthemes, whereas in the plan for analysis, there is mention of subthemes. | Thank you for this observation. We appreciate that some qualitative approaches present themes alongside explicit subthemes. However, our analysis used Thematic Decomposition, in which the primary analytic outputs were three overarching themes. The headings within each theme were used to organise the narrative presentation of the findings rather than to denote formally developed analytical subthemes. To avoid ambiguity, we have revised the analysis section to clarify this aspect of our approach and ensure consistency. |
| How was interview recorded | We have clarified that interviews were audio-recorded with consent and subsequently transcribed verbatim. |
| The first line in discussion mentions that the perspective of HCP in ICCS versus TAU in CAMHS was explored, whereas the findings section describe the perspectives in combination of the two. | Thank you for this helpful suggestion. The primary aim of this qualitative study was to explore healthcare professionals' experiences across the two service models rather than to undertake a formal comparative qualitative analysis. Accordingly, the themes were developed from the dataset as a whole. Nevertheless, to improve clarity, we have strengthened the manuscript by highlighting where particular aspects of care were more strongly emphasised by ICCS participants while identifying themes that were consistently reported across both ICCS and TAU. We have incorporated this comparative synthesis into the Results and Discussion while avoiding interpretations beyond the scope of the data. |
| Some abbreviations are directly mentioned in text like CYP, YP | All abbreviations are now defined at first use. |
| Some references are missing in text - 20, 32, 34, 35 | We have carefully reviewed the manuscript and corrected inconsistencies between in-text citations and the reference list. Missing citations have now been inserted where appropriate. |
| **Reviewer 2 comment** | |
| While the manuscript explains the philosophy of Intensive Community Care Services (ICCS), a more detailed description of the model, including staffing structure, caseloads, intensity of contact, and intervention components, would help readers better understand how ICCS differs from Treatment as Usual (TAU). | Thank you for this helpful suggestion. We have expanded the description of the ICCS model in the Introduction section to provide greater detail on staffing, intervention components, contact intensity, and service delivery. In addition, we have included a summary comparison table that highlights the key distinctions between ICCS and TAU to improve readers' contextual understanding. |
| The paper would benefit from a detailed table that describes the healthcare professionals interviewed, including their profession, years of experience, service type, and geographical location. This would improve transparency and allow readers to better assess transferability. | Thank you for this suggestion. As part of revisions made in response to Reviewer 1, we have added a Participant Characteristics table (Table 2) summarising professional role, years of experience, service type and geographical location. This additional information improves transparency and allows readers to better assess the transferability of the findings. |
| The findings suggest significant emotional labour and risk of burnout among healthcare professionals. A more detailed discussion of workforce wellbeing, staff retention, supervision models, and organisational support mechanisms would strengthen the practical implications. | Thank you for highlighting this important issue. We have expanded the Discussion to consider the emotional labour associated with delivering intensive relational care, together with implications for workforce wellbeing, burnout, supervision and staff retention. We also discuss the importance of organisational support structures, including reflective supervision and protected time for multidisciplinary working, to sustain relational models of care. |
| Many findings focus on relational care across services. A clearer comparison highlighting which themes were unique to ICCS and which were common across ICCS and TAU would improve the manuscript's contribution to service development. | Thank you for this valuable suggestion. The primary aim of the study was to explore healthcare professionals' experiences across ICCS and TAU rather than undertake a formal comparative qualitative analysis. Accordingly, the themes were developed across the combined dataset. Nevertheless, to improve clarity, we have added a comparative synthesis table highlighting experiences common to both services, as well as those more frequently emphasised by ICCS participants. We have also strengthened the Discussion by drawing greater attention to these similarities and differences while remaining consistent with the scope of our analysis. |
| The findings suggest significant emotional labour and risk of burnout among healthcare professionals. A more detailed discussion of workforce wellbeing, staff retention, supervision models, and organisational support mechanisms would strengthen the practical implications. | Thank you for this valuable suggestion. We agree that the implications of emotional labour for workforce wellbeing warrant further consideration.  In response, we have expanded the discussion to examine the implications of providing intensive community-based mental health care for staff wellbeing and service sustainability. The revised manuscript now discusses how the emotionally demanding nature of working within ICCS may contribute to burnout, compassion fatigue and workforce attrition if appropriate organisational supports are not in place. We have integrated evidence demonstrating that sustained emotional demands are associated with exhaustion, disengagement and poorer mental wellbeing among healthcare professionals. |
| Many findings focus on relational care across services. A clearer comparison highlighting which themes were unique to ICCS and which were common across ICCS and TAU would improve the manuscript's contribution to service development. | Thank you for this valuable suggestion. The primary aim of the study was to explore healthcare professionals' experiences across ICCS and TAU rather than undertake a formal comparative qualitative analysis. Accordingly, the themes were developed across the combined dataset. Nevertheless, to improve clarity, we have added a comparative synthesis table highlighting experiences common to both services, as well as those more frequently emphasised by ICCS participants. We have also strengthened the discussion by drawing greater attention to these similarities and differences while remaining consistent with the scope of our analysis. |
| Given increasing pressures on CAMHS services, a discussion regarding the cost-effectiveness, workforce requirements, and scalability of ICCS would be valuable for policymakers and service planners. | Thank you for this suggestion. Although a full economic evaluation was planned for the feasibility trial, a cost-effectiveness analysis couldn’t be conducted due to the sample size. We have expanded the discussion to consider workforce requirements, implementation challenges and the scalability of ICCS. We also acknowledge the need for future economic evaluations alongside effectiveness studies to inform wider service implementation. |
| The manuscript briefly references digital platforms in the literature review but does not explore how technology could support relational care, engagement, and continuity within ICCS services. This may be an important area for future development. | We have expanded the discussion to consider how digital technologies may complement relational care by enhancing communication, continuity of support, and flexible service delivery, while recognising that they should augment rather than replace face-to-face therapeutic relationships. |
| The implications section could provide more concrete recommendations such as:  Recommended caseload sizes for ICCS teams. Minimum training requirements in trauma-informed and relational care. Strategies for reducing waiting times. Frameworks for integrating peer support safely. Models for family psychoeducation and empowerment. | Thank you for this valuable suggestion. We have strengthened the “Implications for Practice” section by providing more explicit recommendations informed by our findings. Specifically, we now discuss the importance of workforce development through training in trauma-informed, relational and family-centred care, the provision of regular clinical supervision and protected opportunities for reflective practice, strengthened multidisciplinary collaboration, flexible models of community-based service delivery, and the integration of family psychoeducation and collaborative care planning into routine practice. As this was a qualitative study, we have intentionally avoided making prescriptive recommendations regarding caseload sizes or specific service standards, as these were beyond the scope of the data. Instead, our recommendations are framed as evidence-informed considerations for future service development, implementation and evaluation. |
| Future studies should:  Evaluate long-term outcomes beyond the intervention period. Examine experiences of families and young people from underserved communities. Investigate the effectiveness of ICCS across different diagnostic groups. Explore implementation strategies for scaling relational care models nationally. | We have expanded the discussion section to outline several priorities for future research arising from the present study. Specifically, we now recommend evaluations that examine the sustainability of outcomes beyond the intervention period; research that explores experiences across diverse communities; implementation research to understand the organisational and contextual factors influencing the successful delivery and scalability of ICCS; and economic evaluations, alongside future effectiveness studies, to inform commissioning and wider service implementation. |

We believe these revisions have substantially improved the manuscript and fully addressed the reviewers' comments.

Once again, we sincerely thank the Editor and reviewers for their careful consideration of our work and for the constructive feedback, which has considerably strengthened the manuscript.

Yours sincerely,

Dr Opeyemi Atanda
