# Supplementary material for: Relational care in community mental health: Evaluating staff experiences in Intensive Community Care Services (ICCS) vs treatment as usual
Source: PLoS One. 2026 Sep 9;21(9):e0356608. doi: 10.1371/journal.pone.0356608 (PMC13557356; doi:10.1371/journal.pone.0356608)
Supplement: Peer review history

# Peer review: Decision Letter 0

**DOI:** 10.1371/journal.pone.0356608.r001

**Authors:** Harikrishnan U

4 Jun 2026

Dear Dr. Atanda,

Thank you for submitting your manuscript to PLOS ONE. After careful consideration, we feel that it has merit but does not fully meet PLOS ONE’s publication criteria as it currently stands. Therefore, we invite you to submit a revised version of the manuscript that addresses the points raised during the review process.

Please submit your revised manuscript by Jul 19 2026 11:59PM. If you will need more time than this to complete your revisions, please reply to this message or contact the journal office at plosone@plos.org. When you're ready to submit your revision, log on to https://www.editorialmanager.com/pone/ and select the 'Submissions Needing Revision' folder to locate your manuscript file.

- A letter that responds to each point raised by the academic editor and reviewer(s). You should upload this letter as a separate file labeled 'Response to Reviewers'.
- A marked-up copy of your manuscript that highlights changes made to the original version. You should upload this as a separate file labeled 'Revised Manuscript with Track Changes'.
- An unmarked version of your revised paper without tracked changes. You should upload this as a separate file labeled 'Manuscript'.

If you would like to make changes to your financial disclosure, please include your updated statement in your cover letter. Guidelines for resubmitting your figure files are available below the reviewer comments at the end of this letter.

If applicable, we recommend that you deposit your laboratory protocols in protocols.io to enhance the reproducibility of your results. Protocols.io assigns your protocol its own identifier (DOI) so that it can be cited independently in the future. For instructions see: https://journals.plos.org/plosone/s/submission-guidelines#loc-laboratory-protocols. Additionally, PLOS ONE offers an option for publishing peer-reviewed Lab Protocol articles, which describe protocols hosted on protocols.io. Read more information on sharing protocols at https://plos.org/protocols?utm_medium=editorial-email&utm_source=authorletters&utm_campaign=protocols.

As the corresponding author, your ORCID iD is verified in the submission system and will appear in the published article. PLOS supports the use of ORCID, and we encourage all coauthors to register for an ORCID iD and use it as well. Please encourage your coauthors to verify their ORCID iD within the submission system before final acceptance, as unverified ORCID iDs will not appear in the published article. *Only*  the individual author can complete the verification step; PLOS staff *cannot*  verify ORCID iDs on behalf of authors.

We look forward to receiving your revised manuscript.

Kind regards,

Harikrishnan U, Ph.D

Academic Editor

PLOS One

Journal Requirements:

When submitting your revision, we need you to address these additional requirements.

1. Please ensure that your manuscript meets PLOS ONE's style requirements, including those for file naming. The PLOS ONE style templates can be found at https://journals.plos.org/plosone/s/file?id=wjVg/PLOSOne_formatting_sample_main_body.pdf and https://journals.plos.org/plosone/s/file?id=ba62/PLOSOne_formatting_sample_title_authors_affiliations.pdf

2. Please expand the acronyms “NIHR HTA” (as indicated in your financial disclosure) so that it states the name of your funders in full.

This information should be included in your cover letter; we will change the online submission form on your behalf.

3. Please remove any funding-related text from the manuscript. Please note that funding information should not appear in any section or other areas of your manuscript. We will publish the funding statement that is present in the Financial Disclosure section of the online submission form.

4. For studies involving third-party data, we encourage authors to share any data specific to their analyses that they can legally distribute. PLOS recognizes, however, that authors may be using third-party data they do not have the rights to share. When third-party data cannot be publicly shared, authors must provide all information necessary for interested researchers to apply to gain access to the data. (https://journals.plos.org/plosone/s/data-availability#loc-acceptable-data-access-restrictions)

For any third-party data that the authors cannot legally distribute, they should include the following information in their Data Availability Statement upon submission:

1) A description of the data set and the third-party source

2) If applicable, verification of permission to use the data set

3) Confirmation of whether the authors received any special privileges in accessing the data that other researchers would not have

4) All necessary contact information others would need to apply to gain access to the data

5. Your ethics statement should only appear in the Methods section of your manuscript. If your ethics statement is written in any section besides the Methods, please delete it from any other section.

If the reviewer comments include a recommendation to cite specific previously published works, please review and evaluate these publications to determine whether they are relevant and should be cited. There is no requirement to cite these works unless the editor has indicated otherwise.

Please review your reference list to ensure that it is complete and correct. If you have cited papers that have been retracted, please include the rationale for doing so in the manuscript text, or remove these references and replace them with relevant current references. Any changes to the reference list should be mentioned in the rebuttal letter that accompanies your revised manuscript. If you need to cite a retracted article, indicate the article’s retracted status in the References list and also include a citation and full reference for the retraction notice.

[Note: HTML markup is below. Please do not edit.]

Reviewers' comments:

Reviewer's Responses to Questions

**Comments to the Author**

1. Is the manuscript technically sound, and do the data support the conclusions?

Reviewer #1: Partly

Reviewer #2: Yes

**********

2. Has the statistical analysis been performed appropriately and rigorously?

Reviewer #1: N/A

Reviewer #2: Yes

**********

3. Have the authors made all data underlying the findings in their manuscript fully available?

The PLOS Data policy

Reviewer #1: No

Reviewer #2: Yes

**********

4. Is the manuscript presented in an intelligible fashion and written in standard English?

Reviewer #1: Yes

Reviewer #2: Yes

**********

Reviewer #1: The manuscript seems promising as it tries to show the HCP experiences of caring for CYP involved in ICCS and TAU or CAMHS treatment. However, the manuscript needs modification and lacks clarity in some areas. Also as stated by the author the data availability is restricted stating sensitive information

1. The aim needs to be clearly stated as the statement of aim is overshadowed by a RCT which was a predecessor

2. In the methodology- sampling technique and how sample size was finalized is lacking

3. The interview guide can be included

4. In the analysis - the method of analysis whether manual or software is not mentioned

5. Socio demographic details are lacking

6. How codes were generated can be described

7. The findings' part includes themes but not subthemes, whereas in the plan for analysis there is mention of subthemes

8. How was interview recorded

9. The first line in discussion mentions that the perspective of HCP in ICCS versus TAU in CAMHS was explored, whereas the findings section describe the perspectives in combination of the two.

10. Some abbreviations are directly mentioned in text like CYP, YP

11. Some references are missing in text - 20, 32, 34, 35

Reviewer #2: While the manuscript explains the philosophy of Intensive Community Care Services (ICCS), a more detailed description of the model, including staffing structure, caseloads, intensity of contact, and intervention components, would help readers better understand how ICCS differs from Treatment as Usual (TAU).

The paper would benefit from a detailed table describing the healthcare professionals interviewed, including profession, years of experience, service type, and geographical location. This would improve transparency and allow readers to better assess transferability.

The findings suggest significant emotional labour and risk of burnout among healthcare professionals. A more detailed discussion of workforce wellbeing, staff retention, supervision models, and organisational support mechanisms would strengthen the practical implications.

Many findings focus on relational care across services. A clearer comparison highlighting which themes were unique to ICCS and which were common across ICCS and TAU would improve the manuscript's contribution to service development.

Given increasing pressures on CAMHS services, a discussion regarding the cost-effectiveness, workforce requirements, and scalability of ICCS would be valuable for policymakers and service planners.

The manuscript briefly references digital platforms in the literature review but does not explore how technology could support relational care, engagement, and continuity within ICCS services. This may be an important area for future development.

The implications section could provide more concrete recommendations such as:

Recommended caseload sizes for ICCS teams.

Minimum training requirements in trauma-informed and relational care.

Strategies for reducing waiting times.

Frameworks for integrating peer support safely.

Models for family psychoeducation and empowerment.

Future studies should:

Evaluate long-term outcomes beyond the intervention period.

Examine experiences of families and young people from underserved communities.

Investigate the effectiveness of ICCS across different diagnostic groups.

Explore implementation strategies for scaling relational care models nationally.

**********

what does this mean?). If published, this will include your full peer review and any attached files.

If you choose “no”, your identity will remain anonymous but your review may still be made public.

**Do you want your identity to be public for this peer review?**  For information about this choice, including consent withdrawal, please see our Privacy Policy

Reviewer #1: No

Reviewer #2: **Yes:**  Dr Anu KN

**********

[NOTE: If reviewer comments were submitted as an attachment file, they will be attached to this email and accessible via the submission site. Please log into your account, locate the manuscript record, and check for the action link "View Attachments". If this link does not appear, there are no attachment files.]

To ensure your figures meet our technical requirements, please review our figure guidelines: https://journals.plos.org/plosone/s/figures

You may also use PLOS’s free figure tool, NAAS, to help you prepare publication quality figures: https://journals.plos.org/plosone/s/figures#loc-tools-for-figure-preparation.

NAAS will assess whether your figures meet our technical requirements by comparing each figure against our figure specifications.

Attachment Submitted filename: PONE-D-26-13555 (1).pdf

# Author comment: Author response to Decision Letter 1

**DOI:** 10.1371/journal.pone.0356608.r002

17 Jul 2026

I have attached a response to reviewers comments.

Attachment Submitted filename: Reviewers comments and responses.docx

# Peer review: Decision Letter 1

**DOI:** 10.1371/journal.pone.0356608.r003

**Authors:** Harikrishnan U

5 Aug 2026

Relational Care in Community Mental Health: Evaluating Staff Experiences in Intensive Community Care Services (ICCS) vs Treatment as Usual.

PONE-D-26-13555R1

Dear Dr. Atanda,

We’re pleased to inform you that your manuscript has been judged scientifically suitable for publication and will be formally accepted for publication once it meets all outstanding technical requirements.

Within one week, you’ll receive an e-mail detailing the required amendments. When these have been addressed, you’ll receive a formal acceptance letter and your manuscript will be scheduled for publication.

An invoice will be generated when your article is formally accepted. Please note, if your institution has a publishing partnership with PLOS and your article meets the relevant criteria, all or part of your publication costs will be covered. Please make sure your user information is up-to-date by logging into Editorial Manager at Editorial Manager® and clicking the ‘Update My Information' link at the top of the page. For questions related to billing, please contact billing support.

If your institution or institutions have a press office, please notify them about your upcoming paper to help maximize its impact. If they’ll be preparing press materials, please inform our press team as soon as possible -- no later than 48 hours after receiving the formal acceptance. Your manuscript will remain under strict press embargo until 2 pm Eastern Time on the date of publication. For more information, please contact onepress@plos.org.

Kind regards,

Harikrishnan U, Ph.D

Academic Editor

PLOS One

Additional Editor Comments (optional):

Reviewers' comments:

Reviewer's Responses to Questions

**Comments to the Author**

Reviewer #1: (No Response)

Reviewer #2: All comments have been addressed

**********

2. Is the manuscript technically sound, and do the data support the conclusions?

Reviewer #1: Yes

Reviewer #2: Yes

**********

3. Has the statistical analysis been performed appropriately and rigorously?

Reviewer #1: N/A

Reviewer #2: Yes

**********

4. Have the authors made all data underlying the findings in their manuscript fully available?

The PLOS Data policy

Reviewer #1: No

Reviewer #2: Yes

**********

5. Is the manuscript presented in an intelligible fashion and written in standard English?

Reviewer #1: Yes

Reviewer #2: Yes

**********

Reviewer #1: All comments have been addressed except one for the ordering of references "Reference 20 is not in sequential order in text"

Reviewer #2: The revision appears to address all substantive reviewer comments. The authors have not only replied to each point but also made corresponding additions to the manuscript (e.g., new comparison tables, expanded methodology, richer discussion, clearer implications, and future research directions).

**********

what does this mean?). If published, this will include your full peer review and any attached files.

If you choose “no”, your identity will remain anonymous but your review may still be made public.

**Do you want your identity to be public for this peer review?**  For information about this choice, including consent withdrawal, please see our Privacy Policy

Reviewer #1: No

Reviewer #2: **Yes:**  Dr Anu KN

**********

# Editor report: Acceptance letter

**DOI:** 10.1371/journal.pone.0356608.r004

**Authors:** Harikrishnan U

PONE-D-26-13555R1

PLOS One

Dear Dr. Atanda,

I'm pleased to inform you that your manuscript has been deemed suitable for publication in PLOS One. Congratulations! Your manuscript is now being handed over to our production team.

At this stage, our production department will prepare your paper for publication. This includes ensuring the following:

* All references, tables, and figures are properly cited

* All relevant supporting information is included in the manuscript submission,

* There are no issues that prevent the paper from being properly typeset

You will receive further instructions from the production team, including instructions on how to review your proof when it is ready. Please keep in mind that we are working through a large volume of accepted articles, so please give us a few days to review your paper and let you know the next and final steps.

Lastly, if your institution or institutions have a press office, please let them know about your upcoming paper now to help maximize its impact. If they'll be preparing press materials, please inform our press team within the next 48 hours. Your manuscript will remain under strict press embargo until 2 pm Eastern Time on the date of publication. For more information, please contact onepress@plos.org.

You will receive an invoice from PLOS for your publication fee after your manuscript has reached the completed accept phase. If you receive an email requesting payment before acceptance or for any other service, this may be a phishing scheme. Learn how to identify phishing emails and protect your accounts at https://explore.plos.org/phishing.

If we can help with anything else, please email us at customercare@plos.org.

Thank you for submitting your work to PLOS One and supporting open access.

Kind regards,

PLOS One Editorial Office Staff

on behalf of

Dr. Harikrishnan U

Academic Editor

PLOS One
